# Cantharidin induces apoptosis of human triple negative breast cancer cells through mir-607-mediated downregulation of EGFR

**DOI:** 10.1186/s12967-023-04483-y

**Published:** 2023-09-05

**Authors:** Tianfeng Yang, Runze Yu, Cheng Cheng, Jian Huo, Zhengyan Gong, Hanbing Cao, Yu Hu, Bingling Dai, Yanmin Zhang

**Affiliations:** 1 School of Pharmacy, Health Science Center, Xi’an Jiaotong University, No. 76, Yanta West Street, #54, Xi’an, 710061 Shaanxi People’s Republic of China; 2 State Key Laboratory of Shaanxi for Natural Medicines Research and Engineering, Xi’an, 710061 People’s Republic of China

**Keywords:** TNBC, Cantharidin, EGFR, miR-607, Apoptosis

## Abstract

**Background:**

Triple negative breast cancer (TNBC) is a major subtype of breast cancer, with limited therapeutic drugs in clinical. Epidermal growth factor receptor (EGFR) is reported to be overexpressed in various TNBC cells. Cantharidin is an effective ingredient in many clinical traditional Chinese medicine preparations, such as Delisheng injection, Aidi injection, Disodium cantharidinate and vitamin B6 injection. Previous studies showed that cantharidin had satisfactory pharmacological activity on a variety of tumors. In this study, we aimed to study the therapeutic potential of cantharidin for TNBC treatment by targeting EGFR, and expound its novel regulator miR-607.

**Methods:**

The effect of cantharidin on breast cancer in vivo was evaluated by 4T1 mice model. Then the effects of cantharidin on TNBC cells was assessed by the MTT, colony formation, and AnnexinV-PE/7AAD staining. Cantharidin acts on EGFR were verified using the cell membrane chromatography, RT-PCR, Western blotting, MTT, and so on. Mechanistic studies were explored by dual-luciferase report assay, RT-PCR, western blotting, and immunofluorescence staining assay.

**Results:**

Cantharidin inhibited TNBC cell growth and induce apoptosis by targeting EGFR. miR-607 was a novel EGFR regulator and exhibited suppressive functions on TNBC cell behaviors. Mechanistic study showed that cantharidin blocked the downstream PI3K/AKT/mTOR and ERK/MAPK signaling pathway.

**Conclusion:**

Our results revealed that cantharidin may be served as a potential candidate for TNBC treatment by miR-607-mediated downregulation of EGFR.

**Graphical Abstract:**

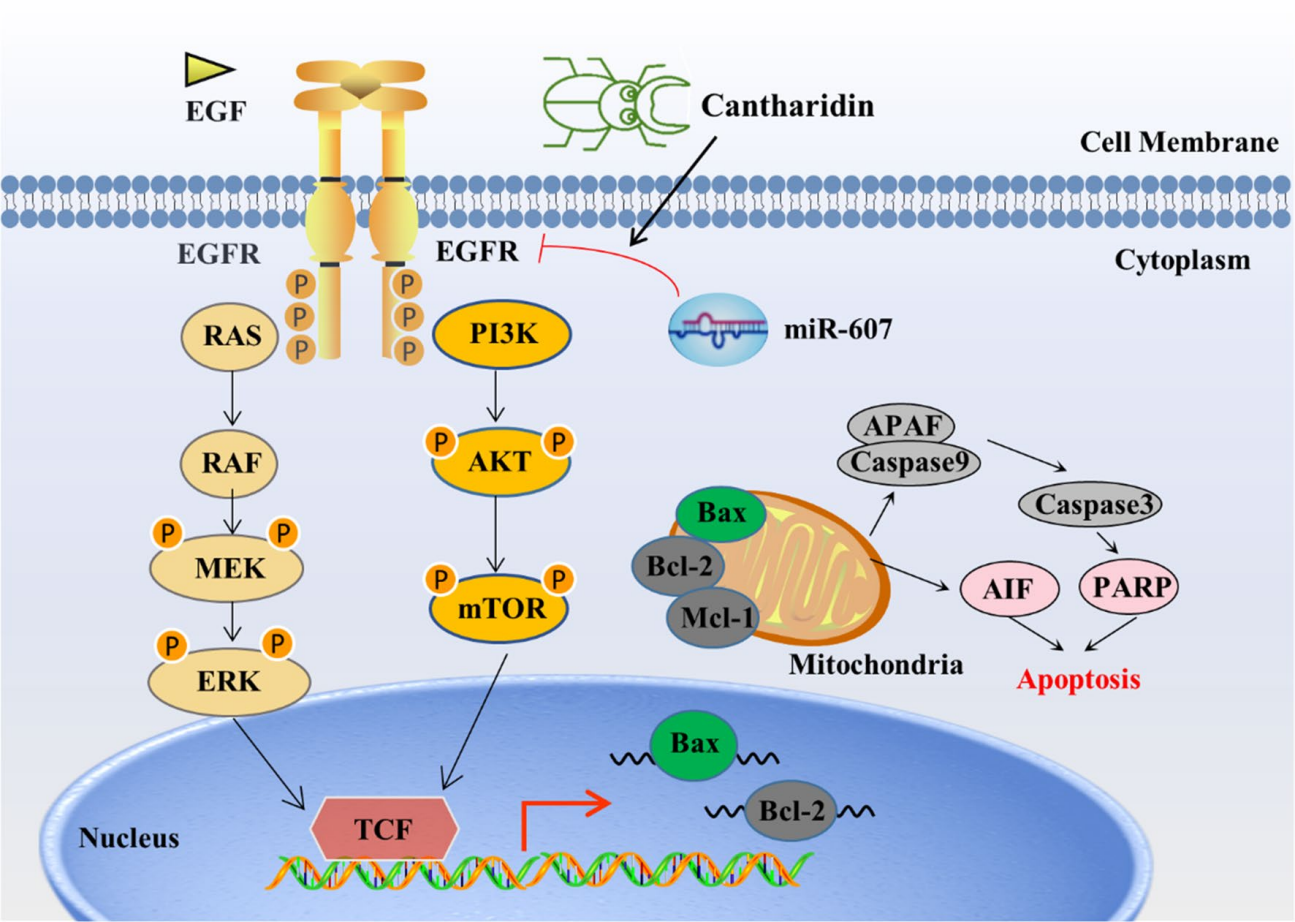

**Supplementary Information:**

The online version contains supplementary material available at 10.1186/s12967-023-04483-y.

## Background

Breast cancer is one of the most prevalent malignant tumors, which has seriously threatened the women health worldwide [1]. Triple negative breast cancer (TNBC), characterized by the absence of estrogen, progesterone and HER2 receptors, is a pernicious molecular subtype of breast cancer and has a more aggressive clinical course [2]. Currently, most patients have a poor prognosis due to the lack of traditionally targeted therapies for TNBC clinical treatment. The treatment of TNBC has become increasingly challenging owing to the highly heterogeneous and the lack of endocrine target and anti-HER2 therapy. Studies have shown that 34% of TNBC patients relapse within 5 years after receiving adjuvant or neoadjuvant chemotherapy [3]. With the development of potential molecular targets in breast cancer, targeted therapy gradually shows its value in specific types. Therefore, it is urgent to explore new and effective therapeutic targeted drugs for TNBC treatment.

Epidermal growth factor receptor (EGFR) has been reported to be abnormally amplified in TNBC cases, which can be served as one of the important therapeutic targets [4]. Activation of EGFR triggers various downstream signaling pathway, such as classical pathways including PI3K/AKT/mTOR, ERK/MAPK, which are involved in cell proliferation, cell cycle progression, primary tumorigenesis and metastasis [5]. At present, a variety of EGFR inhibitors targeting TNBC are used in the clinical treatment of breast cancer, such as the tyrosine kinase inhibitors afatinib, erlotinib and lapatinib, as well as monoclonal antibodies such as cetuximab and panitumumab [6]. The EGFR inhibitor gefitinib inhibits breast cancer cell proliferation and sensitizes cells to carboplatin and docetaxel [7]. The application of EGFR inhibitors alone is not effective, so EGFR inhibitors are often used as adjuvant therapy in clinical practice, in combination with chemotherapy, etc. [8].

MicroRNAs suppress the expression of varieties of genes through binding with their 3′UTRs (3′-untranslated regions), and thus participate in the occurrence and development of TNBC by exerting oncogenic or tumor suppressor properties, and has potential application value [9]. In addition, miRNA relates to the progression, metastasis, prognosis and diagnostic typing of TNBC [10]. Current studies have shown that miR-607 exerts tumor suppressor effects by regulating target genes or proteins, and this process exists in the progression of various malignant tumors including chronic lymphocytic leukemia, cervical cancer and pancreatic cancer, etc. [11–13]. However, there is almost no relevant research on the regulation of TNBC by miR-607.

**Fig. 1:**
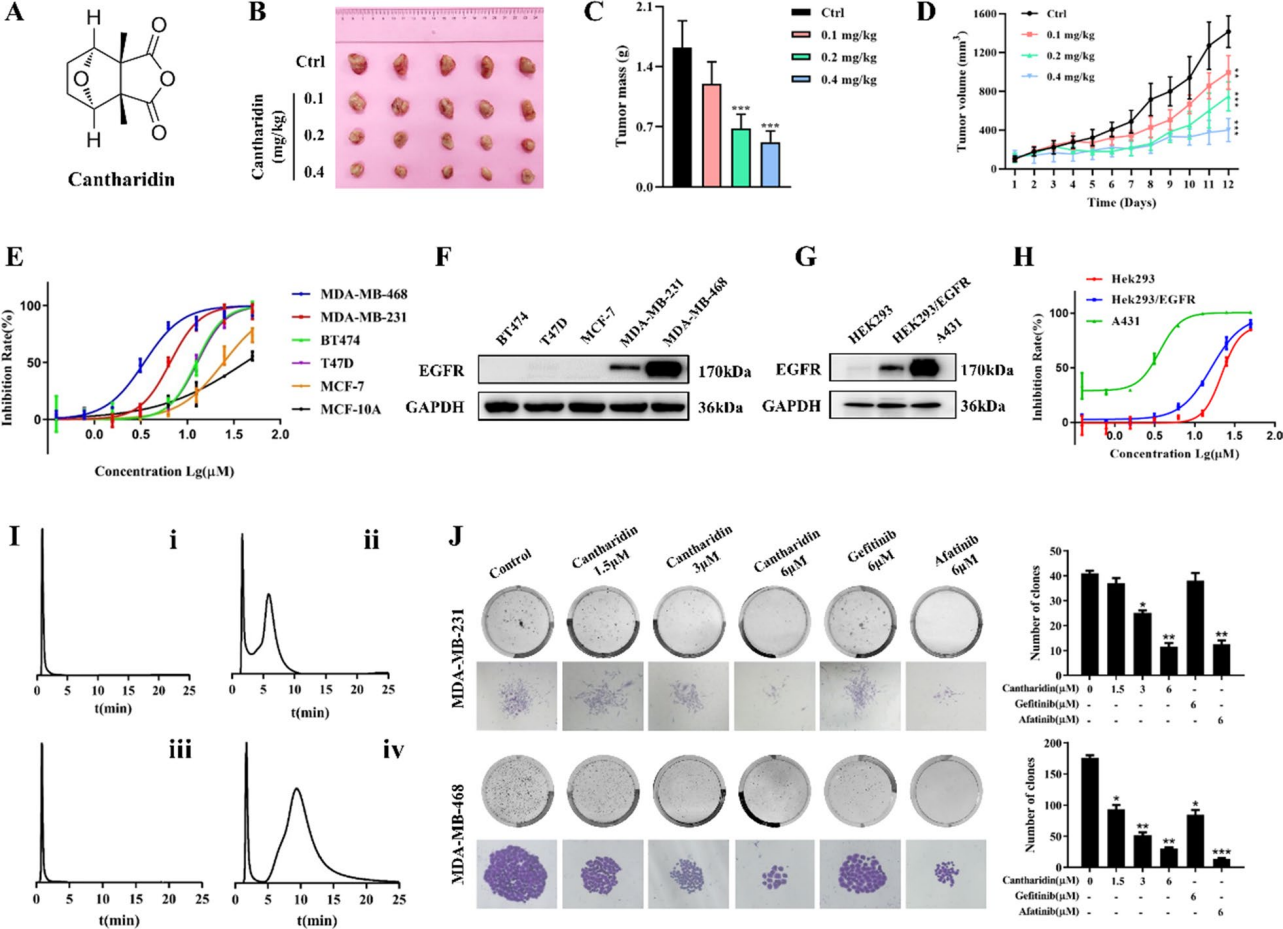
Cantharidin selectively inhibits EGFR-overexpressed cells proliferation. **A** Chemical structure of cantharidin. **B** Images of tumors excised from five nude mice at 12 days after intraperitoneal injection of saline solution or cantharidin in 4T1 homograft tumors. **C** Effects of cantharidin treatment on the masses of 4T1 tumors. **D** Volume changes of 4T1 tumors were measured every day. **E** Effects of cantharidin on cell proliferation in indicated human breast cancer cells and human normal breast epithelial cells MCF-10 A. **F** Protein expression of EGFR in breast cancer cells BT474, T47D, MCF-7, MDA-MB-231 and MDA-MB-468. **G** Protein expression and quantification of EGFR in HEK293, HEK293/EGFR and A431 cells. **H** Effects of cantharidin on cell proliferation in HEK293, HEK293/EGFR and A431 cells. **I** The chromatograms of cantharidin (**i**, **ii**) and afatinib (**iii**, **iv**) on HEK293/CMC (**i**, **iii**) and EGFR/CMC (**ii**, **iv**) column. **J** Effects of cantharidin on the colony formation of MDA-MB-231 and MDA-MB-231 cells. The EGFR inhibitor gefitinib (6 µM) and afatinib (6 µM) were utilized as positive control drug. The colony formation (the upper line) and the individual colony (the lower line) (×200 magnification) were photographed. Data were expressed as mean ± SEM (n = 3), *p < 0.05, **p < 0.01, ***p < 0.001

Cantharides have a long history of being used as traditional Chinese medicine to treat skin diseases, and its active ingredient is mainly cantharidin (Fig. 1A). A variety of Chinese medicine preparations containing cantharidin have achieved good antitumor effect in clinic, such as Delisheng injection, Aidi injection, Disodium cantharidinate and vitamin B6 injection, et al. The antitumor activity of cantharidin and its derivatives can be traced back to 1933, and the studies focused on numerous cancers including breast cancer, leukemia, colon cancer, pancreatic cancer, gastric cancer, lung cancer, melanoma and liver cancer [14]. The multiple mechanisms of cantharidin have been reported: such as inducing autophagy, apoptosis by regulating miR-106b-93/p21-PTEN axis in breast cancer [15], inducing leukemia cell apoptosis by regulating p38 MAPK, JUK, p53 and caspase3 [16], inducing apoptosis in oral squamous cancer cells via the JNK-regualted mitochondria and endoplasmic reticulum stress-related signaling pathways [17], inhibiting cell proliferation and migration in liver cancer by regulating the related proteins of MAPK signaling pathway [18]. In addition, cantharidin can sensitize the effect of radiotherapy on tumor cells [19]. In this study, we explored cantharidin exerts anti-tumor effect by targeting miR-607-regulated EGFR, which provide a new theoretical basis for the further clinical application of cantharidin.

## Methods

### Chemicals and reagents

Cantharidin, Gefitinib, Afatinib and AG1478 were obtained from Meilun Biotech Co., Ltd. (Dalian, China). CCCP was acquired from Solarbio Science & Technology Co., Ltd. (Beijing, China). Dual Luciferase Reporter Assay Kit, miRNA primer and Lipofectamine®3000 were from Vazyme Biotech Co. Ltd. (Nanjing, China). MiR-607 mimic/inhibitor were purchased from General Biol Co., Ltd (Anhui, China). Mir-X miRNA qRT-PCR SYBR Kit and luciferase vector GV306 were bought from Genechem. Co. Ltd. (Shanghai, China). PrimeScript RT Master Mis Perfect kit and SYBR® Premix Ex TaqTM II was obtained from Takara Biomedical Technology Co., Ltd. (Beijing, China). EGFR, p53, Bax, Bcl-2, Mcl-1, mTOR, APAF-1, Caspase9, Cleaved-caspase9, AIF, Caspase3, Cleaved-caspase3, PARP1, GAPDH and Goat anti rabbit IgG were from Protein Technology Group (Chicago, Illinois, USA). p-EGFR, p-AKT, AKT, p-mTOR, p-MEK, MEK, p-ERK, ERK, PI3 Kinase Antibody Sampler Kit (including PI3K-110α, PI3K-110β, PI3K-110γ, p-PI3K-P85/P55, PI3K-P85) and Cleaved-PARP were purchased from Cell Signaling Technology, Inc. (Danvers, Massachusetts, USA). The PE Annexin V Apoptosis Detection Kit I was obtained from BD Biosciences (Franklin lake, New Jersey, USA). FITC-actin and Cy3-goat anti rabbit IgG was purchased from Yeasen Biotechnology.

### Human cell lines and mice

Human breast cancer cell lines BT474 and MDA-MB-468, Human normal mammary epithelial cell MCF-10 A, Human epidermoid carcinoma A431 cells and Mouse breast cancer cell lines 4T1 were purchased from the Shanghai Institute of Cell Biology at the Chinese Academy of Sciences (Shanghai, China). Human breast cancer cell lines T47D, MCF-7 and MDA-MB-231 were purchased from Genechem. Co. Ltd. (Shanghai, China). HEK293 cells were obtained from Professor Xu Li (School of Medicine, Xi’an Jiaotong University). The HEK293/EGFR cell line which overexpresses EGFR was constructed at the Research and Engineering Center for Natural Medicine, Xi’an Jiaotong University.

MDA-MB-231 and MDA-MB-468 cells were cultured in Leibovitz’s L15 medium with 10% (v/v) FBS and 1% P/S; 4T1, BT474, T47D, HEK293 and HEK293/EGFR were cultured in RPMI-1640 medium with 10% (v/v) FBS and 1% P/S; MCF-7 was cultured in DMEM medium with 10% (v/v) FBS and 1% P/S; A431 was cultured in F12 medium with 10% (v/v) FBS medium and 1% P/S. MCF-10 A was cultured in DMEM/F12 with 5% (v/v) HS, 20 ng/mL EGF, 0.5 µg/mL Hydrocortison, 10 µg/mL Insulin, 1% NEAA and 1% P/S. The cell lines were authenticated using STR profiling and tested for mycoplasma contamination. All cell lines were incubated at 37 °C in a humidified incubator containing 5% CO_2_ except for MDA-MB-231 and MDA-MB-468.

BALB/C mice were purchased from GemPharmatech LLC (Nanjing, China) and housed at the Experimental Animal Center of the Xi’an Jiaotong University. The animal procedure was carried out in accordance with National Institute of Health guidelines and Animal Research Committee of Xi’an Jiaotong University. All animal experiments were performed with the permission of the Biomedical Ethics Committee of Xi’an Jiaotong University Health Science Center (Project No. 2019-973, approved on 2019.4.8).

### In vivo tumor suppression assay

4T1 cells were used to establish the tumor homograft model. A total of 2 × 10^6^ cells was subcutaneously injected into the right armpit of mice, the mice were randomly divided into four groups when the tumor was permitted to reach a volume greater than 100 mm^3^. And then, daily intraperitoneal administration of cantharidin (0.1 mg/kg, 0.2 mg/kg and 0.4 mg/kg dissolved in saline solution of 0.5% DMSO) or saline solution of 0.5% DMSO (control group) was done performed for 12 days. The body weights of the mice and tumor volumes were monitored daily. The mice were sacrificed on day 12, and the tumors were removed. All procedures and experiments were approved by the Biomedical Ethics Committee of the Xi’an Jiaotong University Health Science Center.

### Cell viability assay

BT474, T47D, MCF-7, MDA-MB-231, MDA-MB-468, HEK293, HEK293/EGFR, A431, MDA-MB-231-shEGFR, MDA-MB-468-shEGFR, MDA-MB-231-shEGFR + EGFR, MDA-MB-468-shEGFR + EGFR, MCF-7/EGFR cells were seeded and incubated with different concentrations of cantharidin for 48 h. MDA-MB-231 and MDA-MB-468 cells were seeded and incubated with miR-607 NC, miR-607 mimic and miR-607 inhibitor for 12 h, 24 h, 48 and 72 h. MTT solution in serum-free medium was added for another 4 h. After removal of the medium, 150 µL DMSO was added to dissolve the formazan. The absorbance was measured using a microplate reader (Bio-Rad Hercules, CA, USA). The results were expressed as the percentage of cell inhibition rate. The IC_50_ values were calculated using Prism 6.0 GraphPad (La Jolla, CA, USA).

### Colony-forming assay

MDA-MB-231 and MDA-MB-468 cells were seeded and then treated with different concentrations of cantharidin for 48 h. MDA-MB-231 and MDA-MB-468 cells were seeded and incubated with miR-607 NC, miR-607 mimic and miR-607 inhibitor for 24 h. The medium was then replaced with drug-free medium, and the plates were cultured for an additional 10–15 days until the colonies were clearly visible and countable. Colonies were fixed with methanol and stained with crystal violet. Images were obtained using an imaging system (Champchemi Professional, SG2010084, Sage creation, Beijing, China) and an inverted fluorescence microscope (DM505, Nikon Co., Ltd., Otawara, Tochigi, Japan).

### Cell membrane chromatography

CMC analysis was performed on a Shimadzu LC‑20 A apparatus that consisted of two LC‑20AD pumps, a DGU‑20A3 degasser, an SIL‑20 A autosampler, a CTO‑20 A column oven, and an SPD‑M20A diode array detector (Shimadzu, Kyoto, Japan). The data were acquired using LC solution software (Shimadzu). The detection wavelength was 250.4 nm. The chromatographic conditions were as follows: CMC column, 10.0 × 2.0 mm; flow rate, 0.2 mL/min; column temperature, 37 °C; mobile phase, dd H_2_O.

### Lentiviral transfection and construction of stable cell lines

MDA-MB-231 and MDA-MB-468 cells were plated and added the virus vector or control according to the MOI of pre-experiment for transfection. 12 h later, cells were changed with fresh medium and the green fluorescence was observed. The target cells were screened by puromycin and collected for further experiments. ShRNA targeting sequence: GTGGCTGGTTATGTCCTCATT, overexpression EGFR sequence: (NM-005228).

### RNA isolation and RT-PCR

Total RNA from transfected cells was prepared according to the manufacturer’s instructions. Reverse transcription and RT-PCR were performed as described previously [20]. The primer was from General Biol Co., Ltd (Anhui, China). The sequences were listed at Additional file 1: Table S1. 2^−ΔΔCt^ method was used and the amplification curve of β-actin was the same as target gene EGFR, and the amplification curve of U6 was the same as tested miRNAs.

### Dual-luciferase report assay

HEK293T cells were co-transfected with dual-luciferase report gene vector mixed with lipofectamine 3000 and miR-607 mimic in advance for 15 min. Then the cells were rinsed using 1× PBS to perform cell lysis. The supernatant was used to perform Luciferase Assays. To determine the activities of luciferase, Dual Luciferase Reporter Assay Kit was used.

### Immunofluorescence staining assay

MDA-MB-231 and MDA-MB-468 cells treated with cantharidin for 48 h were fixed, blocked, and incubated with a primary antibody against p-ERK (1:150) at 4 °C for 12 h. The cells were then incubated with a Cy3-conjugated secondary antibody (1:50) at 37 ˚C for 2 h. Nuclei were stained with DAPI, and cytoplasm were stained with FITC-labelled phalloidine. Fluorescence images were captured using a Leica TCS SP8 STED 3× laser scanning confocal microscope (Leica, Wetzlar, Germany).

### Flow cytometric analysis of cell apoptosis

Wild-type MDA-MB-231, MDA-MB-468 and MCF-7 cells and constructed EGFR- knockdown or overexpressed cells were treated with cantharidin, CCCP, gefitinib and afatinib, respectively. For miR-607 mimic and inhibitor transfection, the scrambled miRNA was transfected and used as negative control groups. After that, the cells were harvested and stained with 5 µL AnnexinV-PE and 10 µL 7AAD for cell apoptosis analysis. All stained cells were analyzed by FACS (Becton Dickinson, Mountain View, CA, USA). The obtained data was analyzed using Modfit LT software.

### Western blotting

Following stimulation as indicated in different figures, the protein samples were collected and quantified. The insoluble protein lysates were denatured, subjected to SDS-PAGE, transferred to a PVDF membrane, blocked with 5% non-fat milk, incubated with primary antibodies, HRP-conjugated secondary antibodies and visualized using a Tanon 5200 imaging system (Tanon, Shanghai, China). GAPDH was used as a control to normalize protein loading. The Pro Plus software (Image-Pro Plus 5.1, Media Cybernetics, Inc., Rockville, MD, USA) was used for protein quantification.

### Statistical analysis

Quantitative data are expressed as mean ± SEM of three separate experiments for each condition. Statistical analysis was performed using one-way analysis of variance (ANOVA). Tukey’s multiple comparison test was used to analyze statistical differences between groups under different conditions. For other data, an independent sample t test was used. *P-value < 0.05 was considered statistically significant.

## Results

### Cantharidin inhibited the proliferation of breast cells in vitro and in vivo

Firstly, 4T1 homograft nude mice models were established to evaluate the anti-tumor effects of cantharidin in vivo. Figure 1B–D showed that treatment with cantharidin significantly delayed tumor growth, decreased tumor mass and tumor volume. We then analyzed the effects of cantharidin on human breast cell lines including MDA-MB-468, MDA-MB-231, BT474, T47D and MCF-7, cantharidin showed different inhibition on cell viability (Fig. 1E). Meanwhile, MDA-MB-468, MDA-MB-231, BT474, T47D and MCF-7 cells expressed different levels of EGFR (Fig. 1F, Additional file 1: Fig. S1A). Interestingly, cantharidin showed a markedly inhibition on MDA-MB-468 and MDA-MB-231 cells with higher EGFR expression. We then proceeded to investigate the inhibitory effect of cantharidin on HEK293, HEK293/EGFR and A431 cells with different expression of EGFR (Fig. 1G, Additional file 1: Fig. S1B). The results showed that cantharidin inhibited the growth of HEK293, HEK293/EGFR and A431 cells which was correlated with the expression of EGFR (Fig. 1H). We then analyzed the interaction between cantharidin and EGFR by CMC chromatograms. The elution profiles of cantharidin and afatinib for the HEK293/EGFR and HEK293 CMC columns were shown in Fig. 1I. The retention behavior indicated that cantharidin could bind to EGFR like afatinib. These results implied that cantharidin may act inhibition on TNBC included MDA-MB-231 and MDA-MB-468 cells by regulating EGFR.

Meanwhile, cantharidin significantly inhibited MDA-MB-231 and MDA-MB-468 cell colony formation in a concentration-dependent manner using gefitinib and afatinib as controls (Fig. 1J). Furthermore, the apoptosis induced by cantharidin was explored by flow cytometry. As shown in Additional file 1: Fig. S2, after treatment with cantharidin, remarkable increase apoptotic rate cells changed from 4.00 ± 0.56% to 6.15 ± 0.38%, 10.14 ± 0.64%, 21.07 ± 0.65% compared with gefitinib treatment group at 7.14 ± 0.32% and afatinib treatment group at 9.78 ± 0.67% in MDA-MB-231 cells. In MDA-MB-468 cells, the apoptotic rates were 4.23 ± 0.67%, 22.78 ± 1.90%, 62.82 ± 2.10%, and 80.74 ± 4.08% in the control group and cantharidin-treated groups, respectively. And the apoptotic rates were 27.81 ± 1.33% and 82.41 ± 3.42% in gefitinib and afatinib-treated group. However, cantharidin did not induce apoptosis of MCF-7 cells, the apoptotic rates were 2.94 ± 0.49%, 4.73 ± 0.40%, 2.91 ± 0.65%, 4.97 ± 0.49%, 4.24 ± 0.29%, and 11.99 ± 0.64% in the control group, cantharidin-treated groups, gefitinib and afatinib-treated group, respectively. These results indicated that cantharidin inhibited colony formation and induced apoptosis in TNBC cells with high expression of EGFR.

#### EGFR knockdown and overexpression altered cantharidin-indued TNBC cell proliferation inhibition and apoptosis induction

**Fig. 2:**
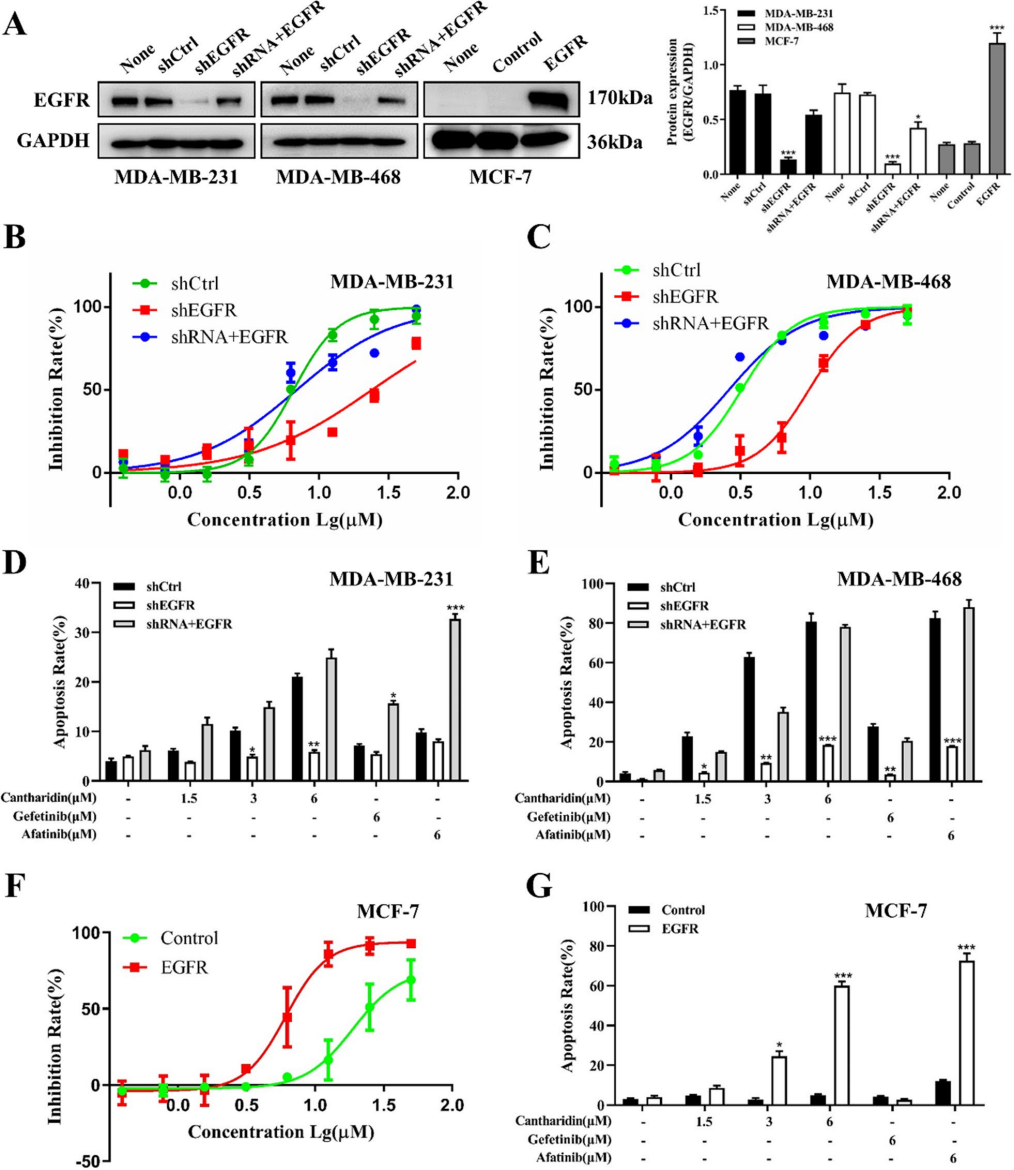
Cantharidin suppresses breast cancer cells growth and induces apoptosis dependent on EGFR levels. **A** Protein levels of EGFR in lentivirus transfected MDA-MB-231, MDA-MB-468 and MCF-7 cells. **B** Effects of cantharidin on cell proliferation in wild-type (shCtrl), EGFR-knockdown (shEGFR) and EGFR-restored (shRNA + EGFR) MDA-MB-231 cells. **C** Effects of cantharidin on cell proliferation in wild-type, EGFR-knockdown and EGFR-restored MDA-MB-468 cells. **D** Effects of cantharidin on cell apoptosis in wild-type, EGFR-knockdown and EGFR-restored MDA-MB-231 cells. **E** Effects of cantharidin on cell apoptosis in wild-type, EGFR-knockdown and EGFR-restored MDA-MB-468 cells. **F** Effects of cantharidin on cell proliferation in wild-type, EGFR-overexpressed MCF-7 cells. **G** Effects of cantharidin on cell apoptosis in wild-type (Control), EGFR-overexpressed (EGFR) MCF-7 cells. Data were expressed as mean ± SEM (n = 3), *p < 0.05, **p < 0.01, ***p < 0.001

To further verify the role of EGFR in the inhibition of TNBC cell proliferation by cantharidin, we knocked down EGFR in MDA-MB-231 and MDA-MB-468 cells, as well as further restoring EGFR levels. Meanwhile, we overexpressed EGFR in MCF-7 cells (Fig. 2A, Additional file 1: Fig. S2). MTT assay showed that EGFR knockdown decreased the sensitivity of MDA-MB-231 and MDA-MB-468 cells to cantharidin, while restoring EGFR rescued the inhibitory effect of cantharidin on MDA-MB-231 and MDA-MB-468 cells (Fig. 2B, C). Also, as shown in Fig. 2D, E, and Additional file 1: Fig. S2A and B, flow cytometry analysis revealed that MDA-MB-231 and MDA-MB-468 apoptosis cells induced by cantharidin were impaired in EGFR-knockdown MDA-MB-231 and MDA-MB-468 cells, while EGFR restoring increased the ratio of apoptotic cells. Moreover, the overexpression of EGFR in MCF-7 cells enhanced the inhibitory effect of cantharidin and increased the apoptotic cells (Fig. 2F, G, Additional file 1: Fig. S2C). These findings confirmed that cantharidin inhibited TNBC cell proliferation and induced apoptosis by regulating EGFR.

### Cantharidin suppressed EGFR activity in TNBC cells

**Fig. 3:**
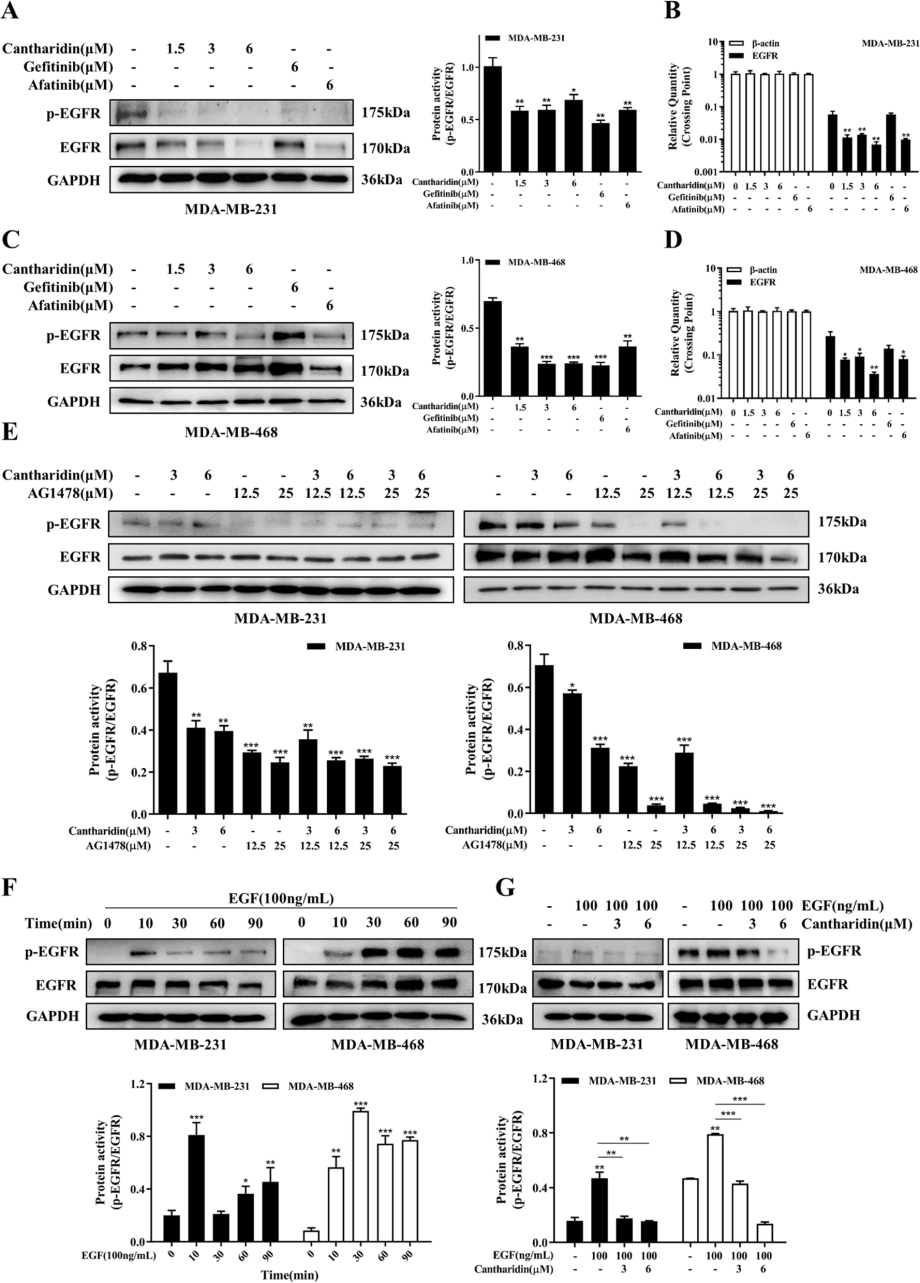
Cantharidin restrains EGFR phosphorylation. **A** Protein expression and quantification of p-EGFR and EGFR in MDA-MB-231 cells at indicated treatment (cantharidin, gefeitinib, afatinib). **B** mRNA level of EGFR in MDA-MB-231 cells at indicated treatment. **C** Protein expression and quantification of p-EGFR and EGFR in MDA-MB-468 cells at indicated treatment. **D** mRNA level of EGFR in MDA-MB-468 cells at indicated treatment. **E** Protein expression and quantification of p-EGFR and EGFR in MDA-MB-231 and MDA-MB-468 cells. The cells were treated with cantharidin or pretreated with EGFR inhibitor AG1478 for 24 h. **F** Protein expression and quantification of p-EGFR and EGFR in MDA-MB-231 and MDA-MB-468 cells. The cells were stimulated by EGF cytokines at different times (10, 30, 60, 90 min). **G** Protein expression and quantification of p-EGFR and EGFR in MDA-MB-231 and MDA-MB-468 cells. The cells were treated with cantharidin or pretreated with EGF in MDA-MB-231 (10 min) and MDA-MB-468 (30 min) cells. Data were expressed as mean ± SEM (n = 3), *p < 0.05, **p < 0.01, ***p < 0.001

To confirm the results of EGFR knockdown and overexpression experiments, we investigated the pharmacological inhibition of EGFR by cantharidin. As shown in Fig. 3A–D, cantharidin not only significantly decreased EGFR mRNA levels but reduced the protein level of EGFR phosphorylation. We also used the AG1478, specific EGFR inhibitor, to further verify the effect of cantharidin on EGFR activity. We found that cantharidin and AG1478 suppressed the EGFR phosphorylation in a concentration manner respectively. Meanwhile, pretreatment with AG1478 before exposure to cantharidin, western blotting results showed that cantharidin could enhance AG1478-induced inhibitory effect (Fig. 3E). Moreover, cantharidin could inhibit the phosphorylation of EGFR stimulated by EGF (Fig. 3F, G). All these results further indicated that cantharidin inhibited EGFR activity.

### Cantharidin blocked PI3K/AKT/mTOR signaling

**Fig. 4:**
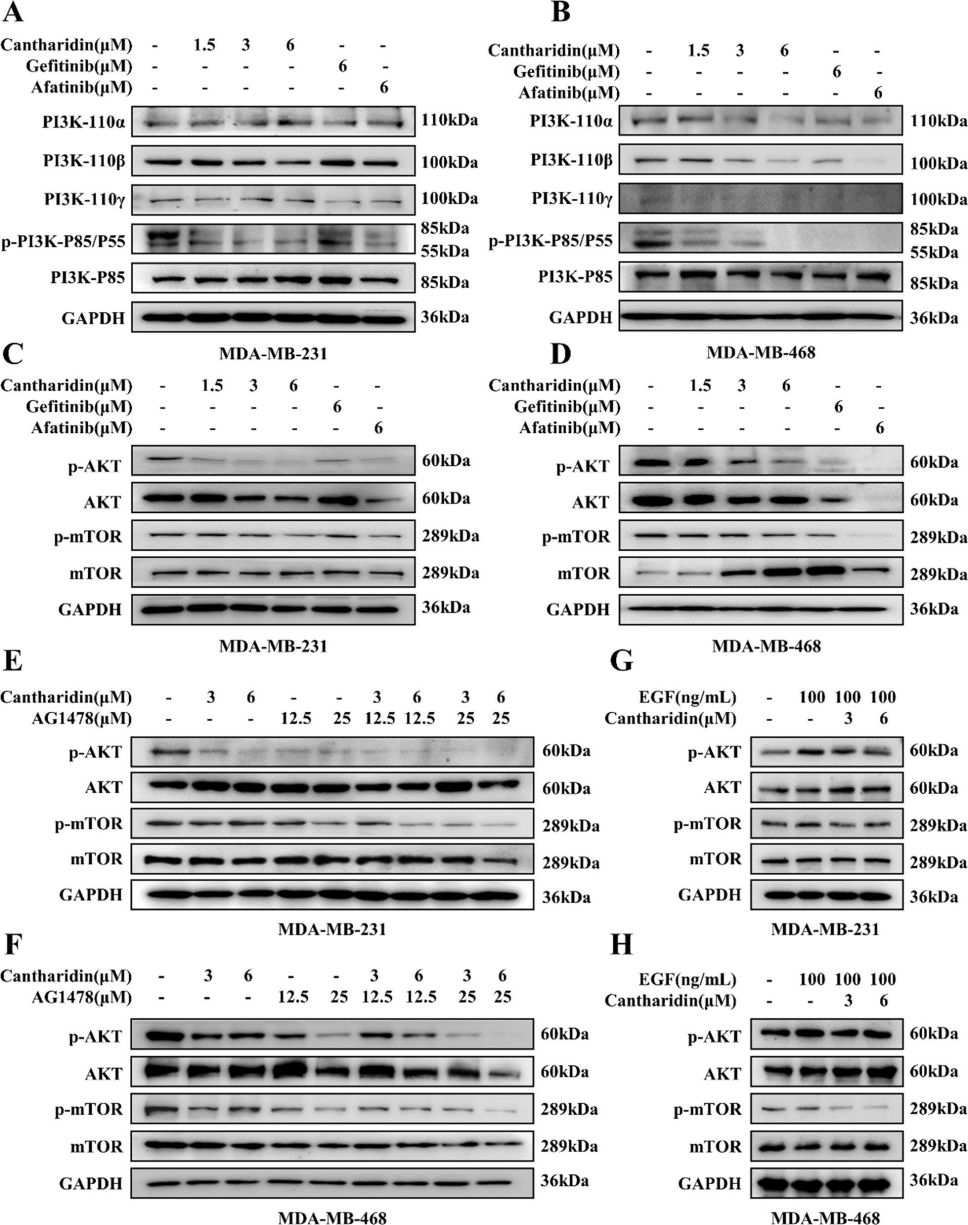
Cantharidin inhibits PI3K/AKT/mTOR signaling pathway. **A** Protein expression of PI3K-110α, PI3K-110β, PI3K-110γ, p-PI3K-P85/55 and PI3K-P85 in MDA-MB-231 cells at indicated treatment. **B** Protein expression of PI3K-110α, PI3K-110β, PI3K-110γ, p-PI3K-P85/55 and PI3K-P85 in MDA-MB-468 cells at indicated treatment. **C** Protein expression of p-AKT, AKT, p-mTOR and mTOR in MDA-MB-231 cells at indicated treatment. **D** Protein expression of p-AKT, AKT, p-mTOR and mTOR in MDA-MB-468 cells at indicated treatment. **E** Protein expression of p-AKT, AKT, p-mTOR and mTOR in MDA-MB-231 cells. The cells were treated with cantharidin or pretreated with EGFR inhibitor AG1478 for 24 h. **F** Protein expression of p-AKT, AKT, p-mTOR and mTOR in MDA-MB-468 cells. The cells were treated with cantharidin or pretreated with EGFR inhibitor AG1478 for 24 h. **G** Protein expression of p-AKT, AKT, p-mTOR and mTOR in MDA-MB-231 cells. The cells were treated with cantharidin or pretreated with EGF for 10 min. **H** Protein expression of p-AKT, AKT, p-mTOR and mTOR in MDA-MB-468 cells. The cells were treated with cantharidin or pretreated with EGF for 30 min

The PI3K/AKT/mTOR signaling pathway is one of the downstream of EGFR and is closely related to the regulation of apoptosis. Therefore, the effect of cantharidin on the PI3K protein molecules and AKT/mTOR were investigated. As shown in Fig. 4A, B, cantharidin inhibited the PI3K-P110β and p-PI3K-P85/P55 subunit in MDA-MB-231 cells and suppressed the PI3K-P110α/β/γ and p-PI3K-P85/P55 in MDA-MB-468 cells (Additional file 1: Fig. S4A, B). Meanwhile, cantharidin downregulated the phosphorylation of AKT and mTOR, the key molecules of the PI3K/AKT signaling pathway in MDA-MB-231 and MDA-MB-468 cells in a concentration-dependent manner (Fig. 4C, D, Additional file 1: Fig. S4C, D). Also, combination of cantharidin and AG1478 showed an enhanced inhibitory effect on AKT and mTOR phosphorylation compared to treatment with either alone in MDA-MB-231 and MDA-MB-468 cells (Fig. 4E, F, Additional file 1: Fig. S4E, F). Moreover, cantharidin suppressed the hyperactivation of EGF-induced AKT and mTOR phosphorylation in MDA-MB-231 and MDA-MB-468 cells (Fig. 4G, H, Additional file 1: Fig. S4G, H).

### Cantharidin disrupted MEK/ERK signaling and induced the intrinsic apoptosis pathway

**Fig. 5:**
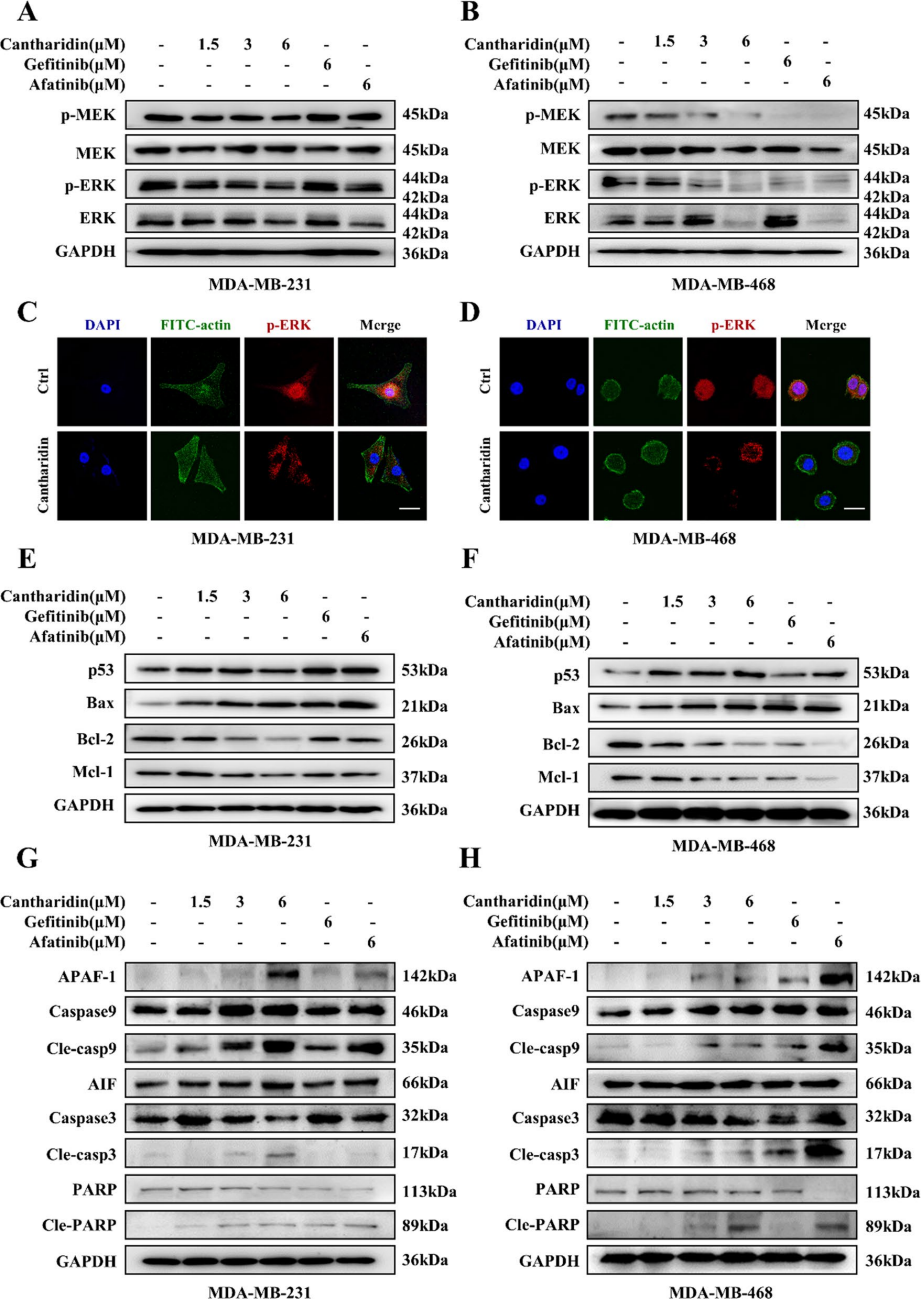
Cantharidin blocks MAPK/ERK signaling and triggers Caspase cascade. **A** Protein expression of p-MEK, MEK, p-ERK and ERK in MDA-MB-231 cells at indicated treatment. **B** Protein expression of p-MEK, MEK, p-ERK and ERK in MDA-MB-468 cells at indicated treatment. Immunofluorescence analysis of p-ERK protein in MDA-MB-231 (**C**) and MDA-MB-468 (**D**) cells treated with cantharidin, p-ERK (red), DAPI (blue) staining, FITC-actin (green) staining and merged images indicate the nuclear translocation and expression of p-ERK, the scale bar represents 20 μm. **E** Protein expression of p53, Bax, Bcl-2 and Mcl-1 in MDA-MB-231 cells at indicated treatment. **F** Protein expression of p53, Bax, Bcl-2 and Mcl-1 in MDA-MB-468 cells at indicated treatment. **G** Protein expression of APAF-1, Caspase9, Cle-casp9, AIF, Caspase3, Cle-casp3, PARP and Cle-PARP in MDA-MB-231 cells at indicated treatment. **H** Protein expression of APAF-1, Caspase9, Cle-casp9, AIF, Caspase3, Cle-casp3, PARP and Cle-PARP in MDA-MB-468 cells at indicated treatment

The MAPK signaling pathway is the other one vital downstream target of EGFR and is involved in the regulation of apoptosis. We also explored the effect of cantharidin on MAPK signaling pathway. As shown in Fig. 5A, B, cantharidin significantly downregulated the phosphorylation of MEK and ERK, the key proteins of the MAPK signaling pathway, in MDA-MB-231 and MDA-MB-468 cells (Additional file 1: Fig. S5A, B). Immunofluorescence staining assay revealed that cantharidin inhibited the nuclear accumulation of p-ERK in the two TNBC cells (Fig. 5C, D).

Subsequently, the expressions of pro-apoptotic proteins including p53 and Bax were upregulated in a concentration-dependent manner, while the levels of the anti-apoptotic protein including Bcl-2 and Mcl-1 were downregulated after cantharidin treatment (Fig. 5E, F, Additional file 1: Fig. S5C, D). Furthermore, with the upregulation of APAF-1 and AIF in cantharidin-exposed cells, the cleavage of Caspase 3, Caspase 9 and PARP were triggered finally (Fig. 5G, H, Additional file 1: Fig. S6).

### MiR-607 was a novel EGFR regulator and its functions on TNBC cell proliferation

**Fig. 6:**
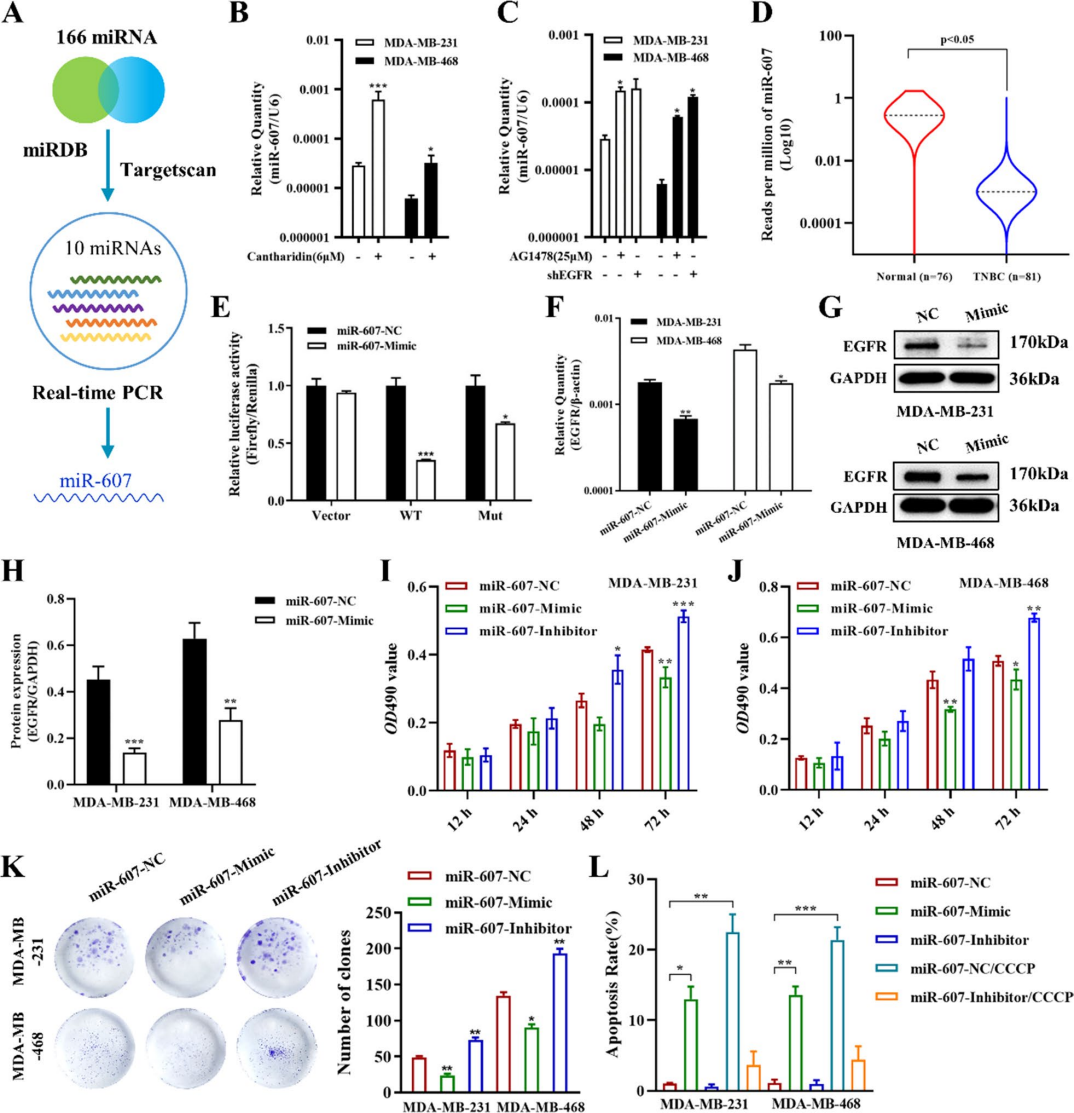
miR-607 is a novel EGFR regulator and upregulated by cantharidin. **A** Schematic diagram of the EGFR-targeted miRNA screening. **B** mRNA level of miR-607 in wild-type and cantharidin-treated MDA-MB-231 and MDA-MB-468 cells. **C** mRNA level of miR-607 in wild-type, AG1478-treated and EGFR-knockdown MDA-MB-231 and MDA-MB-468 cells. **D** The expression of miR-607 was significantly lower in 81 TNBC tumor tissues than in 76 normal breast tissues in the TCGA database. **E** Analysis of the activation of miR-607 in the mild-type and mutant binding sites of EGFR using luciferase reporters in HEK293 cells. mRNA level (**F**) and protein level (**G**) of EGFR in miR-607-NC or miR-607-Mimic transfected MDA-MB-231 and MDA-MB-468 cells. **H** Statistical bar graph of Fig. 6G. Effects of miR-607 on cell proliferation in MDA-MB-231 (**I**) and MDA-MB-468 (**J**) cells by transfected with miR-607-NC, miR-607-Mimic or miR-607-Inhibitor. **K** Effects of miR-607 on the colony formation of MDA-MB-231 and MDA-MB-468 cells by transfected with miR-607-NC, miR-607-Mimic or miR-607-Inhibitor. **L** Effects of miR-607 on CCCP-induced cell apoptosis in MDA-MB-231 and MDA-MB-468 cells by transfected with miR-607-NC, miR-607-Mimic or miR-607-Inhibitor. Data were expressed as mean ± SEM (n = 3), *p < 0.05, **p < 0.01, ***p < 0.001

166 miRNAs that may target EGFR were found through the miRNA target prediction website miRDB, and 10 miRNAs with high targeting coefficients and unreported miRNAs were selected as candidates in combination with miRNA target prediction website TargetScan. The details of 10 miRNAs were as follows: hsa-miR-607, hsa-miR-141-5p, hsa-miR-27a-3p, hsa-miR-27b-3p, hsa-miR-548c-3p, hsa-miR-7-5p, hsa-miR-12,120, hsa-miR-6875-3p, hsa-miR-6888-5p, hsa-miR-3118 (Fig. 6A). After the addition of cantharidin, the contents of miR-607 in MDA-MB-231 and MDA-MB-468 cells was significantly increased (Fig. 6B, Additional file 1: Fig. S7), and the content of miR-607 also increased after treatment with AG1478 and interfering with EGFR expression in cells (Fig. 6C). As shown in Fig. 6D, miR-607 was significantly lower in breast tumor tissue compared with normal breast tissue by TCGA database. It is suggested that miR-607 may play a tumor suppressor role in breast cancer. The miRNA targeting site of EGFR was further verified by dual luciferase reporter gene assay. As shown in Fig. 6E, miR-607 mimic significantly reduced the luciferase activity of the reporter vector, after the mutation in three sites of EGFR 3′-UTR, fluorescence activity was partially offset. Meanwhile, RT-PCR and western blotting results showed that overexpression of miR-607 resulted in a significant decrease in EGFR mRNA and protein expression (Fig. 6F–H). The results indicated that miR-607 is one of the downstream direct regulators of EGFR.

To confirm the role of miR-607 in TNBC, we transfected with miR-607 NC, miR-607 inhibitor, miR-607 mimic, respectively. MTT assay and clone formation results showed that high expression of miR-607 inhibited cell proliferation and clone formation, while low expression of miR-607 played the exact opposite effect (Fig. 6I–K). Meanwhile, Annexin-PE/7AAD double staining kit was used to detect the effect of miR-607 on cell apoptosis. MDA-MB-231 and MDA-MB-468 cells were transfected with miR-607 NC, miR-607 inhibitor, miR-607 mimic, miR-607 NC/CCCP, miR-607 inhibitor/CCCP, respectively. As shown in Fig. 6L, in MDA-MB-231 and 468 cells, the apoptosis rate of NC group was 0.95% and 0.76%, respectively. After transfection of miR-607 Mimic, that is, overexpression of miR-607, the apoptosis rate of both groups was significantly increased, reaching 11.06% and 13.02%, both of which were statistically significant. Meanwhile, transfection with miR-607 inhibitor inhibited the expression of miR-607, the apoptosis rate of the two groups of cells had almost no change; however, miR-607 inhibitor could markedly disturb the apoptosis induced by CCCP (Additional file 1: Fig. S8). All these results showed that upregulated miR-607 promoted MDA-MB-231 and MDA-MB-468 cell apoptosis, which was like the effect of CCCP apoptosis inducer; as well as downregulated miR-607 had the exact opposite effect.

**Fig. 7:**
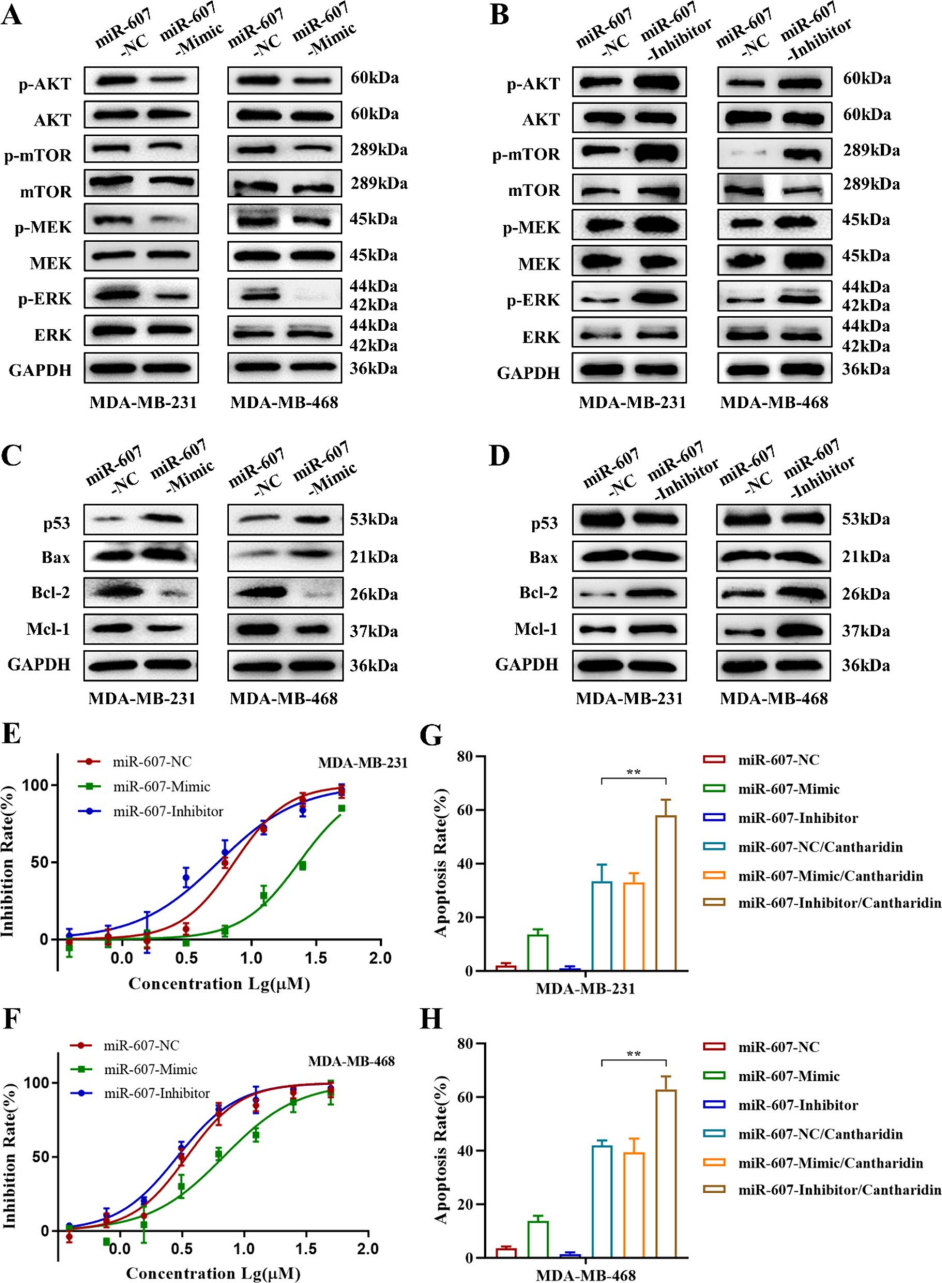
miR-607 regulates EGFR downstream signaling pathway and mediated cantharidin-indued TNBC cell proliferation inhibition and apoptosis induction. **A** Protein expression of p-AKT, AKT, p-mTOR, mTOR, p-MEK, MEK, p-ERK and ERK in miR-607-NC or miR-607-Mimic transfected MDA-MB-231 and MDA-MB-468 cells. **B** Protein expression of p-AKT, AKT, p-mTOR, mTOR, p-MEK, MEK, p-ERK and ERK in miR-607-NC or miR-607-Inhibitor transfected MDA-MB-231 and MDA-MB-468 cells. **C** Protein expression of p53, Bax, Bcl-2 and Mcl-1 in miR-607-NC or miR-607-Mimic transfected MDA-MB-231 and MDA-MB-468 cells. **D** Protein expression of p53, Bax, Bcl-2 and Mcl-1 in miR-607-NC or miR-607-Inhibitor transfected MDA-MB-231 and MDA-MB-468 cells. Effects of cantharidin on cell proliferation in miR-607-NC, miR-607-Mimic or miR-607-Inhibitor transfected MDA-MB-231 (**E**) and MDA-MB-468 (**F**) cells. Effects of cantharidin on cell apoptosis in miR-607-NC, miR-607-Mimic or miR-607-Inhibitor transfected MDA-MB-231 (**G**) and MDA-MB-468 (**H**) cells. Data were expressed as mean ± SEM (n = 3), **p < 0.01

The effect of miR-607 on EGFR downstream signaling pathway AKT/mTOR and MER/ERK was investigated by western blotting to clarify the regulatory mechanism of miR-607 in TNBC. The results indicated that high expression of miR-607 downregulated the phosphorylation levels of AKT, mTOR, MEK and ERK (Fig. 7A, Additional file 1: Fig. S9A, C). Also, low expression of miR-607 upregulated these proteins activity (Fig. 7B, Additional file 1: Fig. S9B, D). Furthermore, overexpressed miR-607 upregulated the apoptosis-related proteins p53 and Bax expression, downregulated the apoptosis-related proteins Bcl-2 and Mcl-1 expression (Fig. 7C, Additional file 1: Fig. S9E, F), while low expressed miR-607 exhibited the opposite effect (Fig. 7D, Additional file 1: Fig. S9G, H).

### Cantharidin-induced apoptosis of TNBC cells was mediated by miR-607

Then we evaluated the effect of cantharidin of MDA-MB-231 and MDA-MB-468 cells in the presence of miR-607-mimic or inhibitor. MTT assay and flow cytometry results showed that cantharidin exerted more inhibition on cell proliferation and induced cell apoptosis more significantly in miR-607-inhibitor transfected cells (Fig. 7E–H, Additional file 1: Fig. S10). These results further verified cantharidin inhibited cell proliferation and induced cell apoptosis by miR-607-mediated downregulation of EGFR.

## Discussion

EGFR, as a molecular target, has crucial role in cancer treatment. Here, we showed that cantharidin exerted anti-proliferation effect and induced apoptosis in TNBC cells by targeting EGFR. Furthermore, we identified miR-607, which was constitutively expressed in breast cancer, as a suppressor of EGFR. MiR-607 directly targets EGFR and attenuates its oncogenic effects in vitro. Moreover, we verified that cantharidin could induce apoptosis of TNBC cells by regulating miR-607-mediated downregulation of EGFR. Our findings suggest that cantharidin may be a potential candidate for TNBC therapy.

Breast cancer is one of the most common malignancies worldwide [21]. Triple negative breast cancer (TNBC) is an important molecular subtype of breast cancer, and there are limited effective treatments and therapeutic strategies for TNBC, thus there is an urgent need to find effective target drugs for TNBC [22]. Previous studies have shown that cantharidin has a good pharmacological activity on a variety of tumors [23]. In this study, we found that cantharidin inhibited 4T1 homograft nude mice growth in vivo. Meanwhile, cantharidin had potent antiproliferative effect on human breast cell lines and showed different inhibitory effect which was related to the EGFR expression. Cantharidin showed more suppressive effect on MDA-MB-231 and MDA-MB-468 cells, which are triple-negative breast cancer cells with high abundance of EGFR. Furthermore, cantharidin was found to had a greater inhibition on A431 and HEK293/EGFR cells with higher EGFR levels. The EGFR/HEK293, HEK293 cell membrane chromatography results revealed that cantharidin could bind to EGFR. These results suggested that cantharidin may exert an inhibitory effect on TNBC cells including MDA-MB-231 and MDA-MB-468 cells by regulating EGFR. In addition, cantharidin also inhibited colony formation and exerted an apoptotic induction effect on MDA-MB-231 and MDA-MB-468 cells, which have high EGFR expression levels.

To further investigate the role of EGFR in cantharidin inhibitory effect in breast cancer cells. We then carried out the lentivector transfection to stably downregulate EGFR in high EGFR-expressing MDA-MB-231 and MDA-MB-468 cells, as well as further restoring EGFR levels. Meanwhile, low EGFR-expressing MCF-7 cells were selected for stable upregulation of EGFR. MTT and flow cytometry results showed that EGFR knockdown alleviated the effect of cantharidin on the proliferation and apoptosis of MDA-MB-231 and MDA-MB-468 cells, while restoring EGFR rescued the inhibitory effect and increased the apoptosis induction by cantharidin. Conversely, overexpression of EGFR significantly exacerbated the effect of cantharidin on MCF-7 cells. To confirm the results of EGFR knockdown and overexpression experiments, we investigated the pharmacological inhibition of EGFR by cantharidin. RT-PCR and western blot results showed that cantharidin downregulated the transcription of mRNA and decreased the phosphorylation of EGFR. The EGFR inhibitor AG1478 and EGF stimulation were also used to further confirm the specific target of cantharidin. The results indicated that cantharidin combined with AG1478 showed an enhanced inhibitory effect on EGFR phosphorylation, also cantharidin decreased the phosphorylation of EGFR stimulated by EGF.

The PI3K/AKT/mTOR signaling pathway is one of the downstream of EGFR and is closely related to the regulation of apoptosis. When activated by PI3K, phosphorylated AKT induces a series of downstream signaling events related to tumor cell growth and apoptosis [24]. Our data presented that cantharidin downregulated PI3K subunit, and inhibited the phosphorylation of AKT and mTOR. Cantharidin could augment the inhibitory effect of AG1478 on AKT and mTOR phosphorylation. Notably, cantharidin inhibited AKT and mTOR phosphorylation induced by EGF implied that the PI3K/AKT pathway by cantharidin occurred via EGFR inhibition. In addition to the PI3K/AKT/mTOR signaling pathway, the ERK/MAPK signaling pathway has been shown to be regulated by EGFR [25]. We also observed cantharidin treatment decreased the phosphorylation of MEK and ERK, as well as inhibited the nuclear localization of p-ERK in MDA-MB-231 and MDA-MB-468 cells. Activation of mTOR and ERK is widely associated with anti-apoptotic functions by binding to transcription factors of Bcl-2 family that downregulate pro-apoptotic proteins and upregulate anti-apoptotic proteins level [26]. Subsequently, the dysfunction of mitochondria induces intrinsic apoptosis through Caspase cascade [27]. Moreover, we verified that cantharidin regulated the apoptotic related proteins and induced the intrinsic apoptosis pathway. These findings implied that cantharidin exerted its inhibitory effect possibly through blocking the activity of EGFR.

10 candidate miRNAs that may target EGFR were predicted through the miRDB and Targetscan. RT-PCR results revealed that miR-607 was upregulated by cantharidin. Dual-luciferase activity assay was used to verify EGFR was one of the downstream target gene of miR-607. RT-PCR and western blotting results further indicated that miR-607 overexpression lead to a decrease of EGFR mRNA and protein. In addition, miR-607 upregulated in AG1478 cells and shEGFR treated cells. These results speculated that cantharidin may play a role through downregulating EGFR medicated by miR-607. Transfected with miR-607 mimic and miR-607 inhibitor in MDA-MB-231 and MDA-MB-468 cells, MTT and colony formation results showed that high expression of miR-607 inhibited cell proliferation and colony formation. Annexin-PE/7AAD staining assay showed that high expression of miR-607 induced MDA-MB-231 and MDA-MB-468 cell apoptosis, playing an effect like CCCP, an apoptosis inducer. Conversely, low expression of miR-607 exhibited the opposite biological function. Western blotting results analyzed that high expression of miR-607 downregulated the phosphorylation levels of AKT, mTOR, MEK, and ERK; in addition, affected the apoptosis-related proteins p53, Bax, Bcl-2, and Mcl-1. On the contrary, miR-607 downregulation had the opposite effect on the expression of these proteins. Furthermore, MTT and flow cytometry results further verified cantharidin exerted more inhibition on cell proliferation and more apoptosis induction when transfected with miR-607-inhibitor. It was further confirmed that cantharidin exerted biological function through upregulating miR-607.

## Conclusions

The present study indicated that miR-607 was a novel EGFR regulator and its functions on TNBC cell proliferation and apoptosis. Cantharidin markedly inhibited TNBC cell proliferation and induced cell apoptosis by targeting EGFR medicated by miR-607. Moreover, mechanistic studies showed that cantharidin inhibited downstream PI3K/AKT/mTOR, ERK/MAPK events, and apoptosis-related proteins. Our findings suggest that cantharidin has therapeutic potential and can serve as an inhibitor in TNBC.

### Supplementary Information

**Additional file 1: Figure S1.** **A** Statistical bar graph of Fig. 1E. **B** Statistical bar graph of Fig. 1G. Data were expressed as mean ± SEM (n = 3). **Figure S2.** **A** Representative results of cell apoptosis in wild-type, EGFR-knockdown and EGFR-restored MDA-MB-231 cells at indicated treatment. **B** Representative results of cell apoptosis in wild-type, EGFR-knockdown and EGFR-restored MDA-MB-468 cells at indicated treatment. **C** Representative results of cell apoptosis in wild-type and EGFR-overexpressed MCF-7 cells. **Figure S3.** mRNA levels of EGFR in lentivirus transfected MDA-MB-231 (**A**), MDA-MB-468 (**B**) and MCF-7 (**C**) cells. Data were expressed as mean ± SEM (n = 3), *p < 0.05, **p < 0.01. **Figure S4.** Statistical bar graph of Fig. 4. Data were expressed as mean ± SEM (n = 3), *p < 0.05, **p < 0.01, ***p < 0.001. **Figure S5.** **A**, **B** Statistical bar graph of Fig. 5A, B. **C**, **D** Statistical bar graph of Fig. 5E, F. Data were expressed as mean ± SEM (n = 3), *p < 0.05, **p < 0.01, ***p < 0.001. **Figure S6.** **A**, **B** Statistical bar graph of Fig. 5G. **C**, **D** Statistical bar graph of Fig. 5H. Data were expressed as mean ± SEM (n = 3), *p < 0.05, **p < 0.01, ***p < 0.001. **Figure S7.** mRNA level of miR-141-5p, miR-27a-3p, miR-27b-3p and miR-548c-3p in MDA-MB-231 (**A**) and MDA-MB-468 (**B**) cells. mRNA level of miR-7-5p, miR-12120, miR-6875-3p, miR-6888-5p and miR-3118 in MDA-MB-231 (**C**) and MDA-MB-468 (**D**) cells. Data were expressed as mean ± SEM (n = 3), *p < 0.05. **Figure S8.** Representative results of miR-607 on CCCP-induced cell apoptosis in MDA-MB-231 (**A**) and MDA-MB-468 (**B**) cells by transfected with miR-607-NC, miR-607-Mimic or miR-607-Inhibitor. **Figure S9.** **A**, **C** Statistical bar graph of Fig. 7A. **B**, **D** Statistical bar graph of Fig. 7B. **E**, **F** Statistical bar graph of Fig. 7C. **G**, **H** Statistical bar graph of Fig. 7D. Data were expressed as mean ± SEM (n = 3), **p < 0.01, ***p < 0.001. **Figure S10.** Representative results of cantharidin-induced cell apoptosis in miR-607-NC, miR-607-Mimic or miR-607-Inhibitor transfected MDA-MB-231 (**A**) and MDA-MB-468 (**B**) cells. **Table S1.** The primers have been used for RT-PCR.

## Data Availability

Data are available on request from the authors. The data that support the findings of this study are available from the corresponding author, Y.M. Zhang., upon reasonable request.

## Abbreviations

CMC: Cell membrane chromatography
DMEM: Dulbecco’s modified eagle medium
DMSO: Dimethyl sulfoxide
ECL: Enhanced chemiluminescence
EGFR: Epidermal growth factor receptor
FBS: Fetal bovine serum
HRP: Horseradish peroxidase
MEM: Minimum essential medium
MTT: 3-(4,5-Dimethy lthiazol-2-yl)-2,5-diphenyltetrazolium bromide
PI: Propidium iodide
PVDF: Polyvinylidene difluoride membrane
RNase: Ribonuclease
SDS-PAGE: Sulfate-polyacrylamide gel electrophoresis
TNBC: Triple negative breast cancer

## References

[1] Siegel RL (2021). Cancer statistics, 2021. CA Cancer J Clin.

[2] Denkert C (2017). Molecular alterations in triple-negative breast cancer-the road to new treatment strategies. Lancet.

[3] Bianchini G (2016). Triple-negative breast cancer: challenges and opportunities of a heterogeneous disease. Nat Rev Clin Oncol.

[4] You KS (2021). Potentiating therapeutic effects of epidermal growth factor receptor inhibition in triple-negative breast cancer. Pharmaceuticals.

[5] Schlessinger J (2000). Cell signaling by receptor tyrosine kinases. Cell.

[6] Balko JM (2012). Profiling of triple-negative breast cancers after neoadjuvant chemotherapy identifies targetable molecular alterations in the treatment-refractory residual disease. Cancer Res.

[7] Balko JM (2012). Profiling of residual breast cancers after neoadjuvant chemotherapy identifies DUSP4 deficiency as a mechanism of drug resistance. Nat Med.

[8] Loibl S (2018). Addition of the PARP inhibitor veliparib plus carboplatin or carboplatin alone to standard neoadjuvant chemotherapy in triple-negative breast cancer (BrighTNess): a randomised, phase 3 trial. Lancet Oncol.

[9] Malla RR (2019). A perspective on the diagnostics, prognostics, and therapeutics of microRNAs of triple-negative breast cancer. Biophys Rev.

[10] Koleckova MM (2018). MicroRNAs in triple-negative breast cancer. Neoplasma.

[11] Chen Y (2019). miR27b3p and miR607 cooperatively regulate BLM gene expression by directly targeting the 3′UTR in PC3 cells. Mol Med Rep.

[12] Xia L (2018). Circular RNA circ-CBFB promotes proliferation and inhibits apoptosis in chronic lymphocytic leukemia through regulating miR-607/FZD3/Wnt/beta-catenin pathway. Biochem Biophys Res Commun.

[13] Wu B (2022). LncRNA LINC00115 facilitates lung cancer progression through miR-607/ITGB1 pathway. Environ Toxicol.

[14] Wang GF (2018). Overview of cantharidin and its analogues. Curr Med Chem.

[15] Li HC (2017). Cantharidin inhibits the growth of triple-negative breast cancer cells by suppressing autophagy and inducing apoptosis in vitro and in vivo. Cell Physiol Biochem.

[16] Su CC (2016). Cantharidin induced oral squamous cell carcinoma cell apoptosis via the JNK-regulated mitochondria and endoplasmic reticulum stress-related signaling pathways. PLoS ONE.

[17] Chen YJ (2009). A small-molecule metastasis inhibitor, norcantharidin, downregulates matrix metalloproteinase-9 expression by inhibiting Sp1 transcriptional activity in colorectal cancer cells. Chem Biol Interact.

[18] Lei AP (2016). Cantharidin inhibits cell proliferation and induces apoptosis through G2/ME phase cell cycle arrest in hepatocellular carcinoma stem cells. Oncol Rep.

[19] Xu MD (2018). The radiotherapy-sensitization effect of cantharidin: mechanisms involving cell cycle regulation, enhanced DNA damage, and inhibited DNA damage repair. Pancreatology.

[20] Yang TF (2018). Novel compounds TAD-1822-7-F2 and F5 inhibited HeLa cells growth through the JAK/Stat signaling pathway. Biomed Pharmacother.

[21] Chen X (2018). Cyclin E overexpression sensitizes triple-negative breast cancer to wee1 kinase inhibition. Clin Cancer Res.

[22] Kalimuth M (2015). Targeted therapies for triple-negative breast cancer: combating a stubborn disease. Trends Pharmacol Sci.

[23] Bertini I (2009). Structural basis of serine/threonine phosphatase inhibition by the archetypal small molecules cantharidin and norcantharidin. J Med Chem.

[24] LoRusso PM (2016). Inhibition of the PI3K/AKT/mTOR pathway in solid tumors. J Clin Oncol.

[25] Roberts PJ (2007). Targeting the Raf-MEK-ERK mitogen-activated protein kinase cascade for the treatment of cancer. Oncogene.

[26] Zhang G (2019). beta-Thujaplicin induces autophagic cell death, apoptosis, and cell cycle arrest through ROS-mediated Akt and p38/ERK MAPK signaling in human hepatocellular carcinoma. Cell Death Dis.

[27] Wu H (2021). mTOR activation initiates renal cell carcinoma development by coordinating ERK and p38MAPK. Cancer Res.

