# Supplementary material for: Cantharidin induces apoptosis of human triple negative breast cancer cells through mir-607-mediated downregulation of EGFR
Source: J Transl Med. 2023 Sep 5;21:597. doi: 10.1186/s12967-023-04483-y (PMC10481602; doi:10.1186/s12967-023-04483-y)
Supplement: Supplementary file 1 — Additional file 1: Figure S1. A Statistical bar graph of Fig. 1E. B Statistical bar graph of Fig. 1G. Data were expressed as mean ± SEM (n = 3). Figure S2. A Representative results of cell apoptosis in wild-type, EGFR-knockdown and EGFR-restored MDA-MB-231 cells at indicated treatment. B Representative results of cell apoptosis in wild-type, EGFR-knockdown and EGFR-restored MDA-MB-468 cells at indicated treatment. C Representative results of cell apoptosis in wild-type and EGFR-overexpressed MCF-7 cells. Figure S3. mRNA levels of EGFR in lentivirus transfected MDA-MB-231 (A), MDA-MB-468 (B) and MCF-7 (C) cells. Data were expressed as mean ± SEM (n = 3), *p < 0.05, **p < 0.01. Figure S4. Statistical bar graph of Fig. 4. Data were expressed as mean ± SEM (n = 3), *p < 0.05, **p < 0.01, ***p < 0.001. Figure S5. A, B Statistical bar graph of Fig. 5A, B. C, D Statistical bar graph of Fig. 5E, F. Data were expressed as mean ± SEM (n = 3), *p < 0.05, **p < 0.01, ***p < 0.001. Figure S6. A, B Statistical bar graph of Fig. 5G. C, D Statistical bar graph of Fig. 5H. Data were expressed as mean ± SEM (n = 3), *p < 0.05, **p < 0.01, ***p < 0.001. Figure S7. mRNA level of miR-141-5p, miR-27a-3p, miR-27b-3p and miR-548c-3p in MDA-MB-231 (A) and MDA-MB-468 (B) cells. mRNA level of miR-7-5p, miR-12120, miR-6875-3p, miR-6888-5p and miR-3118 in MDA-MB-231 (C) and MDA-MB-468 (D) cells. Data were expressed as mean ± SEM (n = 3), *p < 0.05. Figure S8. Representative results of miR-607 on CCCP-induced cell apoptosis in MDA-MB-231 (A) and MDA-MB-468 (B) cells by transfected with miR-607-NC, miR-607-Mimic or miR-607-Inhibitor. Figure S9. A, C Statistical bar graph of Fig. 7A. B, D Statistical bar graph of Fig. 7B. E, F Statistical bar graph of Fig. 7C. G, H Statistical bar graph of Fig. 7D. Data were expressed as mean ± SEM (n = 3), **p < 0.01, ***p < 0.001. Figure S10. Representative results of cantharidin-induced cell apoptosis in miR-607-NC, miR-607-Mimic or miR-607-Inhibitor transfecte [file 12967_2023_4483_MOESM1_ESM.docx]

Cantharidin induces apoptosis of human triple negative breast cancer cells through miR-607-mediated downregulation of EGFR

Tianfeng Yang ^1, 2^, Runze Yu ^1, 2^, Cheng Cheng ^1, 2^, Jian Huo ^1 2^, Zhengyan Gong ^1, 2^, Hanbing Cao ^1, 2^, Yu Hu ^1, 2^, Bingling Dai ^1, 2, *^, Yanmin Zhang ^1, 2, *^

^1^ School of Pharmacy, Health Science Center, Xi’an Jiaotong University, Xi’an 710061, P. R. China

^2^ State Key Laboratory of Shaanxi for Natural Medicines Research and Engineering Xi’an 710061 P.R. China

**Correspondence to:**

Dr. Bingling Dai and Yanmin Zhang

School of Pharmacy, Health Science Center, Xi’an Jiaotong University, No. 76, Yanta Weststreet, #54, Xi’an, Shaanxi Province 710061, P.R. China

Tel.: +86 29 8265 6264; fax: +86 29 8265 5451

E-mail addresses: [dbl1412@xjtu.edu.cn](mailto:dbl1412@xjtu.edu.cn) (BL. Dai)

zhang2008@mail.xjtu.edu.cn (YM. Zhang)

**
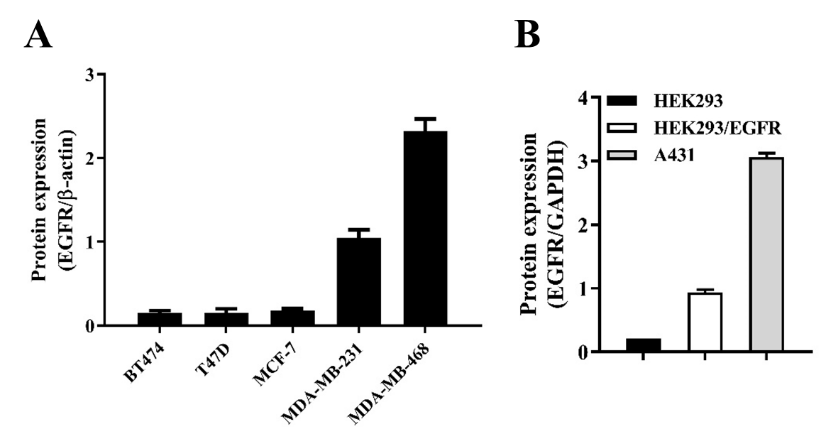
**

**Fig. S1**

**Fig. S1. A** Statistical bar graph of Fig. 1E. **B** Statistical bar graph of Fig. 1G. Data were expressed as mean ± SEM (n = 3).

**
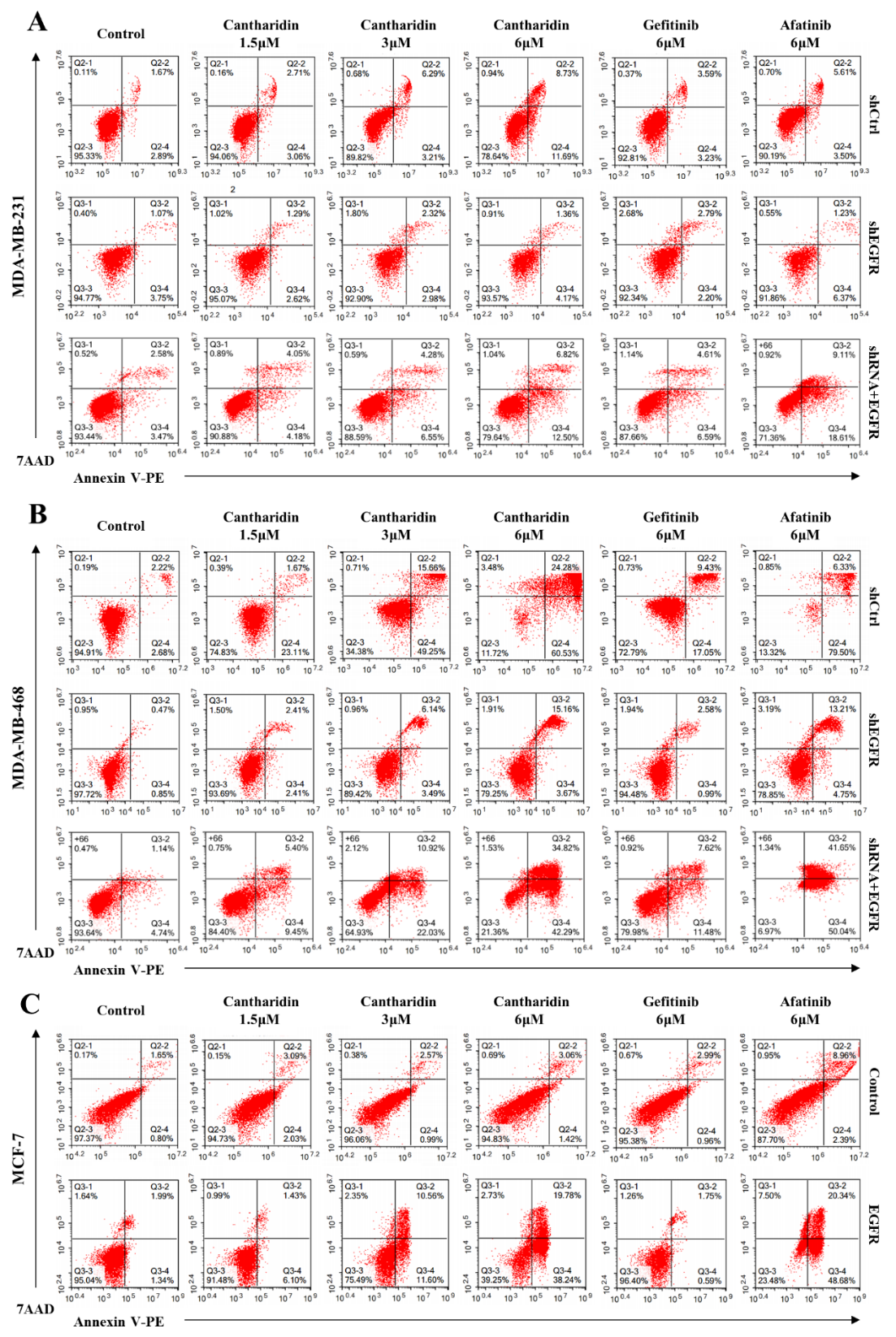
**

**Fig. S2**

**Fig. S2. A** Representative results of cell apoptosis in wild-type, EGFR-knockdown and EGFR-restored MDA-MB-231 cells at indicated treatment. **B** Representative results of cell apoptosis in wild-type, EGFR-knockdown and EGFR-restored MDA-MB-468 cells at indicated treatment. **C** Representative results of cell apoptosis in wild-type and EGFR-overexpressed MCF-7 cells.


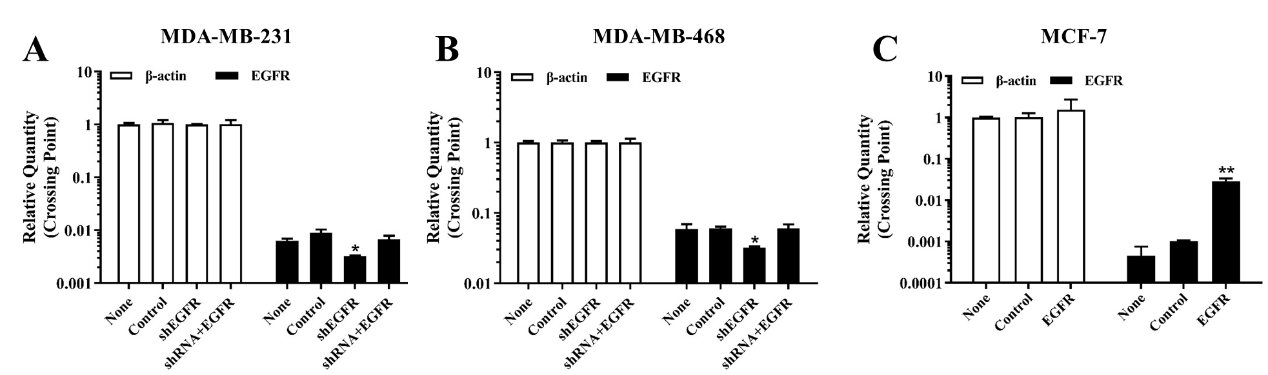


**Fig. S3**

**Fig. S3.** mRNA levels of EGFR in lentivirus transfected MDA-MB-231 (**A**), MDA-MB-468 (**B**) and MCF-7 (**C**) cells. Data were expressed as mean ± SEM (n = 3), **p* < 0.05, ***p* < 0.01.


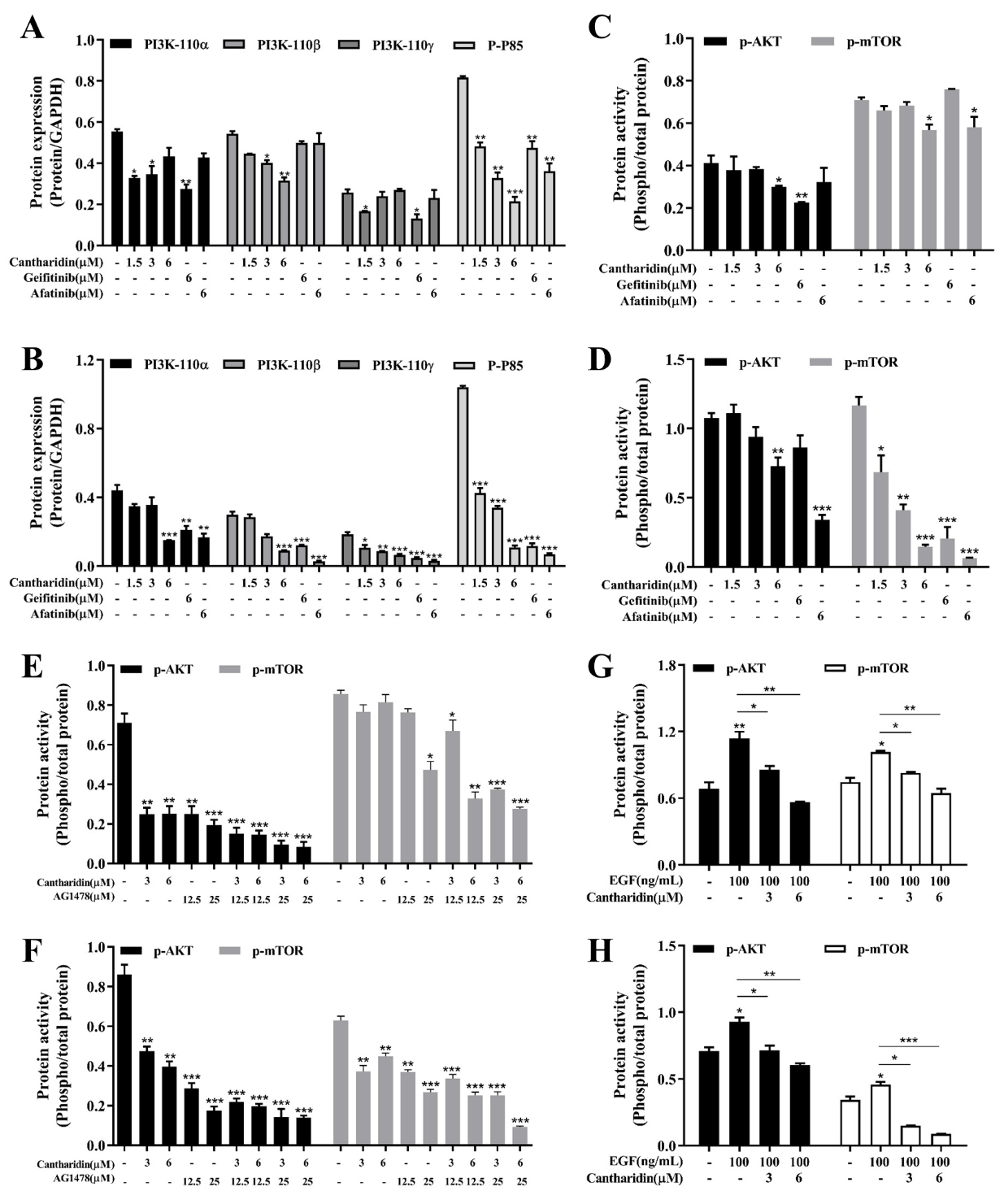


**Fig. S4**

**Fig. S4.** Statistical bar graph of Fig. 4. Data were expressed as mean ± SEM (n = 3), **p* < 0.05, ***p* < 0.01, ****p* < 0.001.


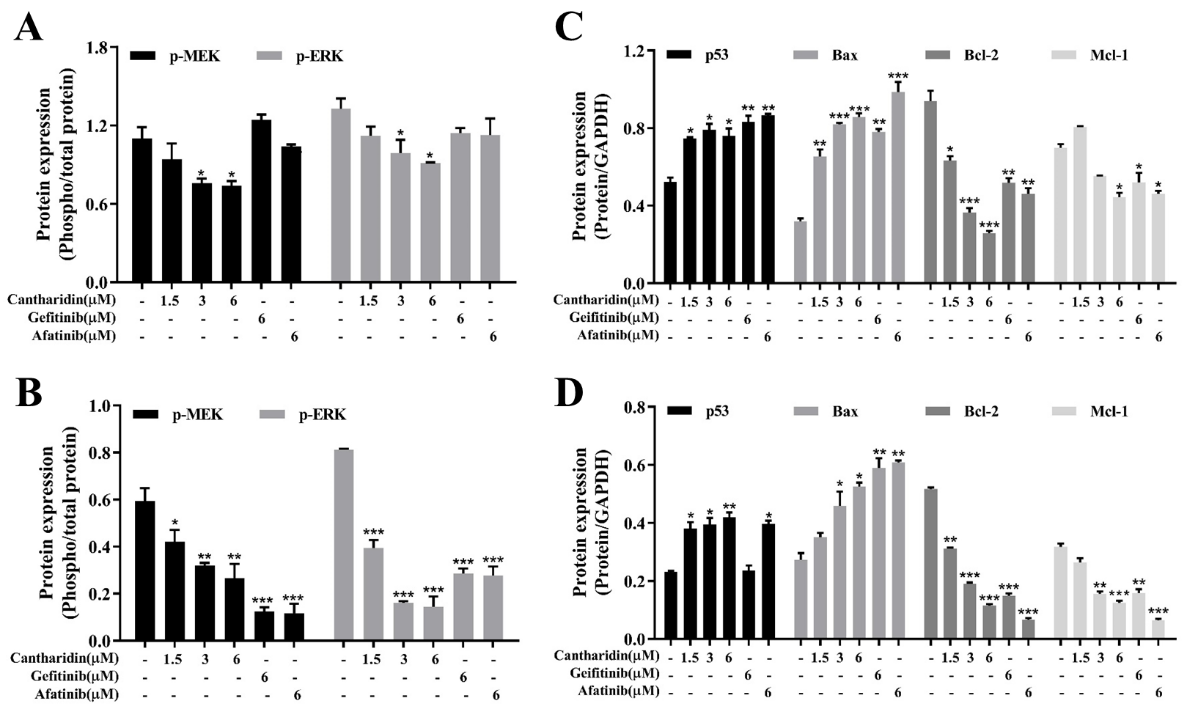


**Fig. S5**

**Fig. S5. A**, **B** Statistical bar graph of Fig. 5A, B. **C**, **D** Statistical bar graph of Fig. 5E, F. Data were expressed as mean ± SEM (n = 3), **p* < 0.05, ***p* < 0.01, ****p* < 0.001.


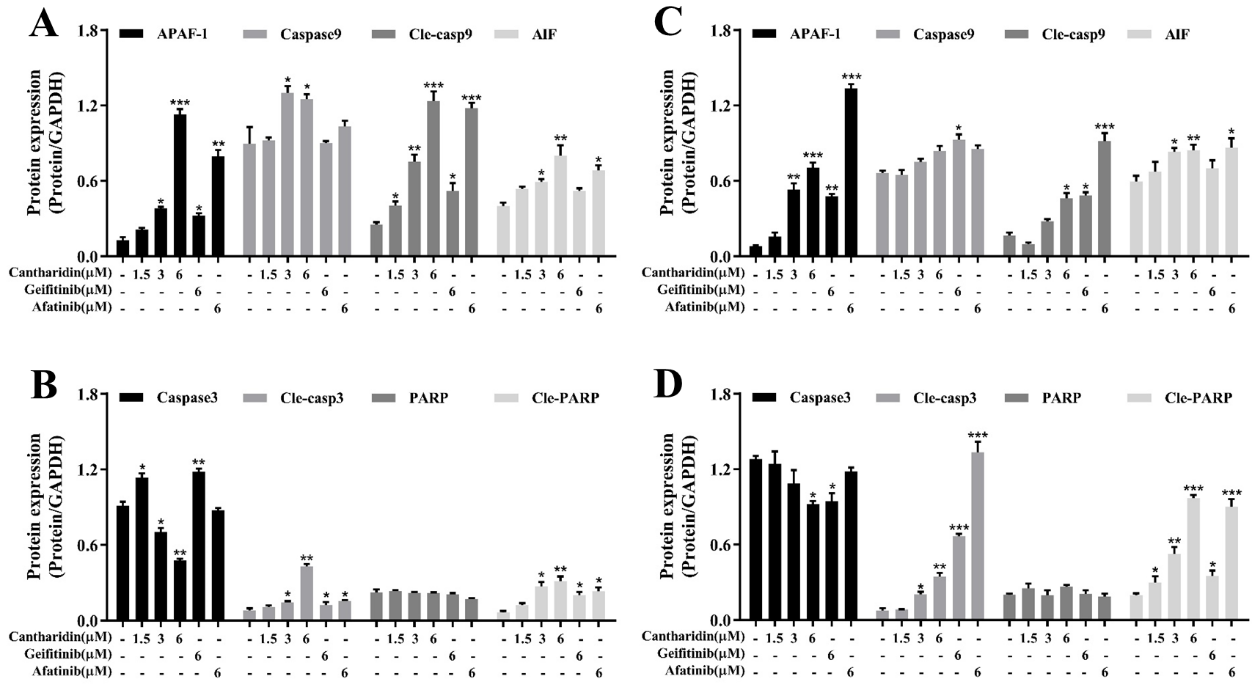


**Fig. S6**

**Fig. S6. A**, **B** Statistical bar graph of Fig. 5G. **C**, **D** Statistical bar graph of Fig. 5H. Data were expressed as mean ± SEM (n = 3), **p* < 0.05, ***p* < 0.01, ****p* < 0.001.


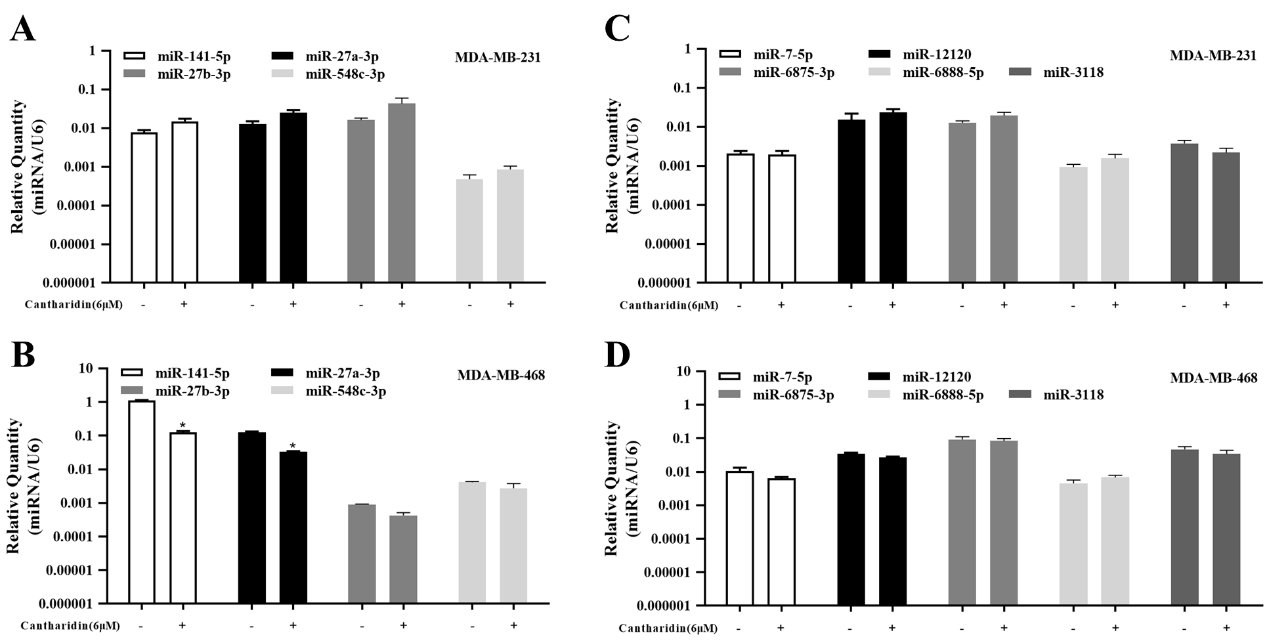


**Fig. S7**

**Fig. S7.** mRNA level of miR-141-5p, miR-27a-3p, miR-27b-3p and miR-548c-3p in MDA-MB-231 (**A**) and MDA-MB-468 (**B**) cells. mRNA level of miR-7-5p, miR-12120, miR-6875-3p, miR-6888-5p and miR-3118 in MDA-MB-231 (**C**) and MDA-MB-468 (**D**) cells. Data were expressed as mean ± SEM (n = 3), **p* < 0.05.


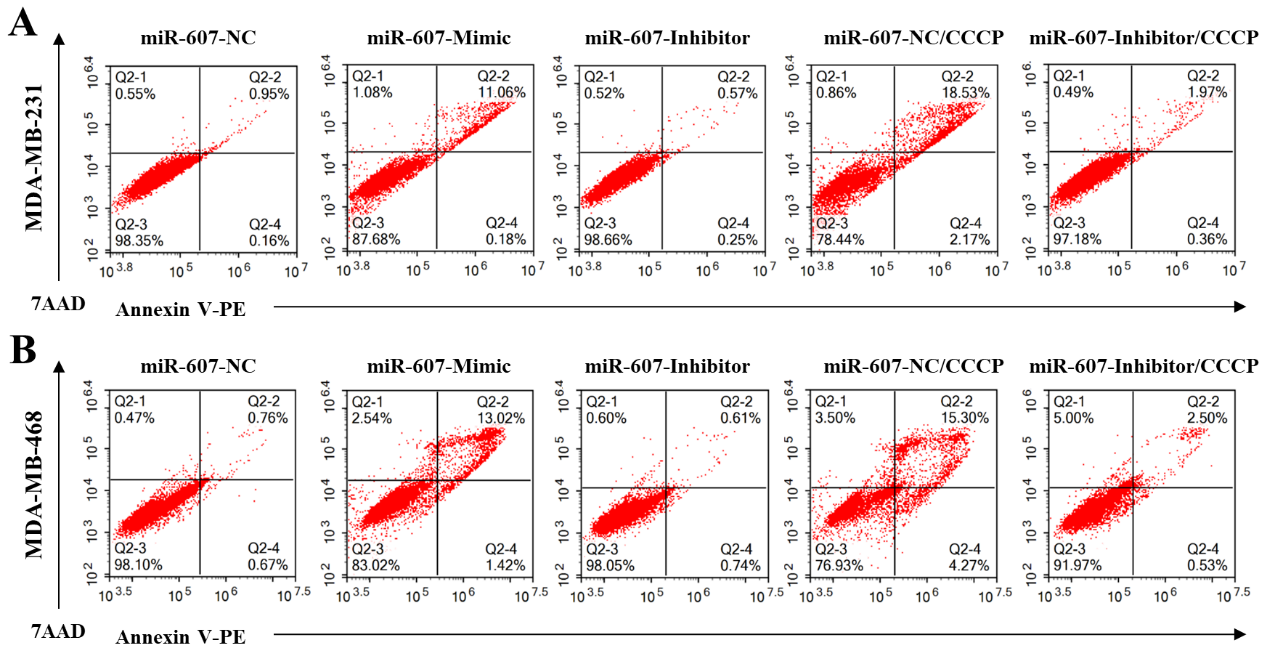


**Fig. S8**

**Fig. S8.** Representative results of miR-607 on CCCP-induced cell apoptosis in MDA-MB-231 (**A**) and MDA-MB-468 (**B**) cells by transfected with miR-607-NC, miR-607-Mimic or miR-607-Inhibitor.


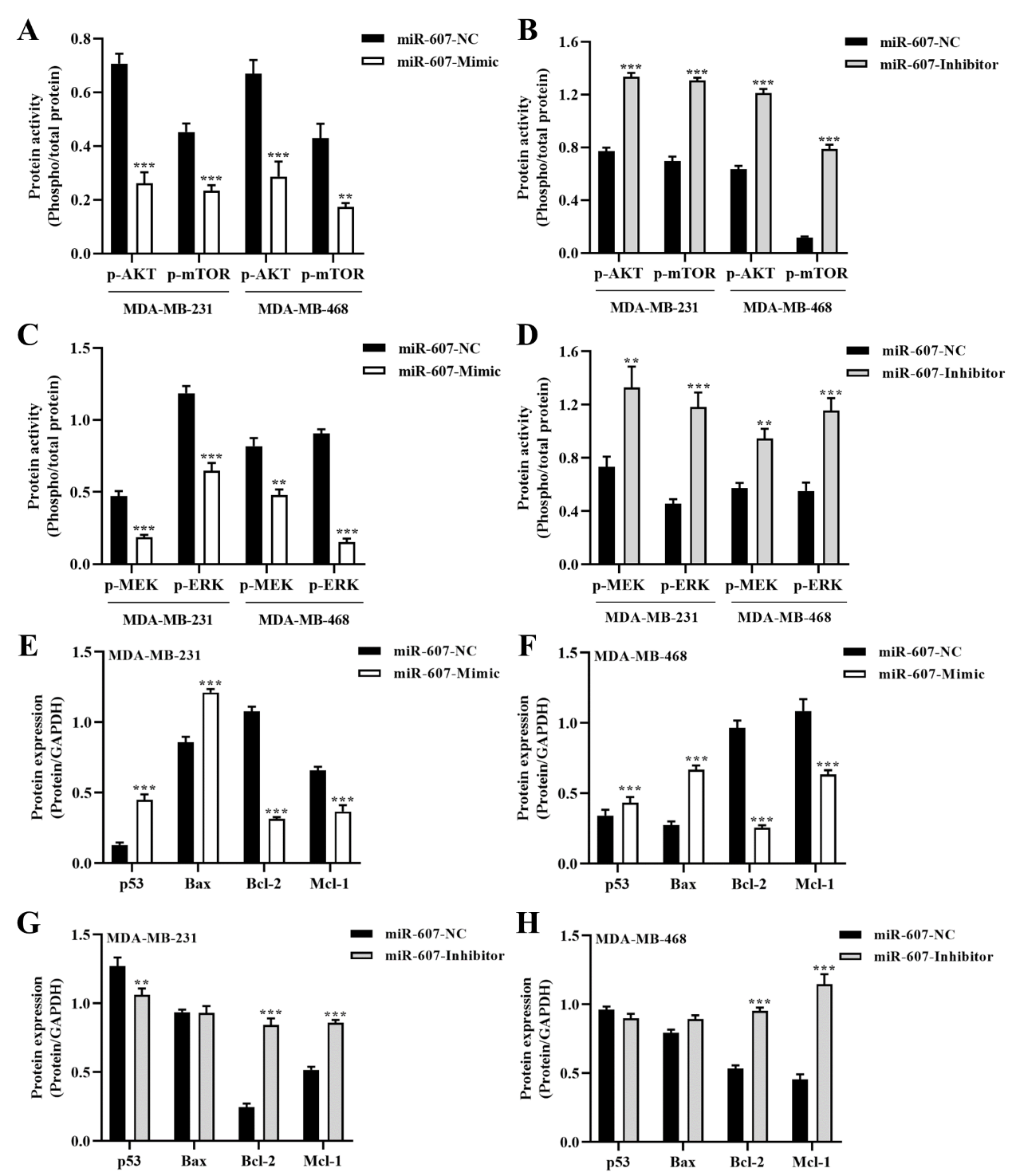


**Fig. S9**

**Fig. S9. A**, **C** Statistical bar graph of Fig. 7A. **B**, **D** Statistical bar graph of Fig. 7B. **E**, **F** Statistical bar graph of Fig. 7C. **G**, **H** Statistical bar graph of Fig. 7D. Data were expressed as mean ± SEM (n = 3), ***p* < 0.01, ****p* < 0.001.


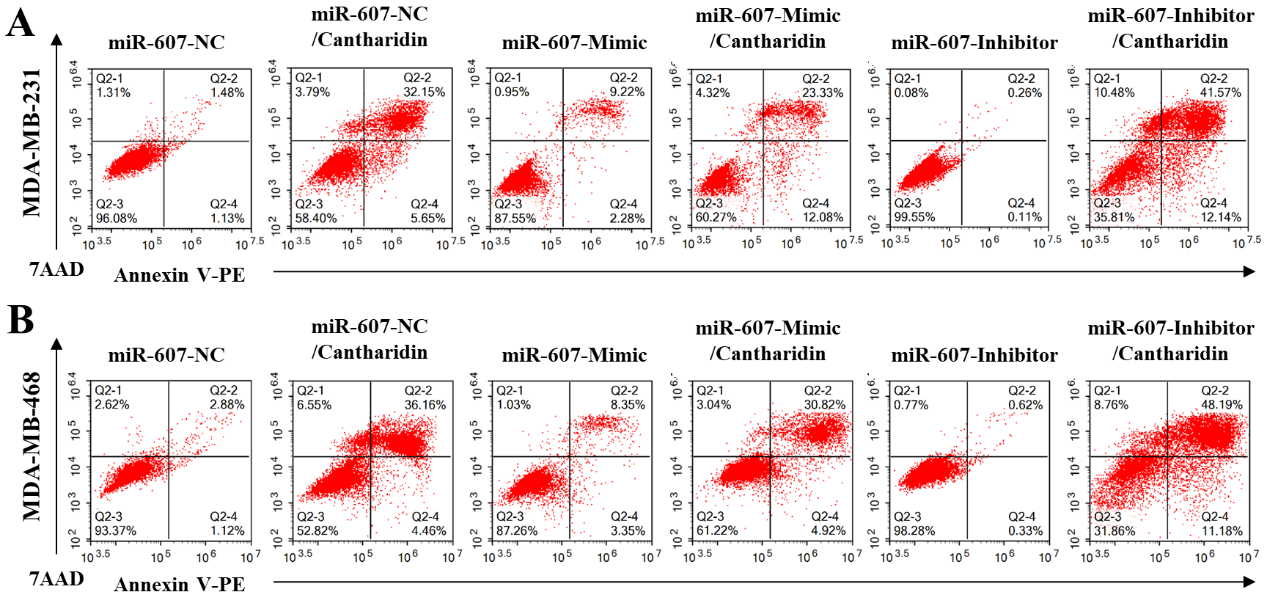


**Fig. S10**

**Fig. S10.** Representative results of cantharidin-induced cell apoptosis in miR-607-NC, miR-607-Mimic or miR-607-Inhibitor transfected MDA-MB-231 **(A)** and MDA-MB-468 **(B)** cells.

**Table S1. The primers have been used for RT-PCR.**

| **Gene/miRNA** | | **Primer sequences** |
| --- | --- | --- |
| EGFR-F | 5’-TGCATACAGTGCCACCCAGAG-3’ | |
| EGFR-R | 5’-GCACACTGGATACAGTTGTCTGGTC-3’ | |
| β-actin-F | 5’-GCGTGACATTAAGGAGAAG-3’ | |
| β-actin-R | 5’-GAAGGAAGGCTGGAAGAG-3’ | |
| miR-607 | GUUCAAAUCCAGAUCUAUAAC | |
| miR-141-5p | CAUCUUCCAGUACAGUGUUGGA | |
| miR-27a-3p | UUCACAGUGGCUAAGUUCCGC | |
| miR-27b-3p | UUCACAGUGGCUAAGUUCUGC | |
| miR-548c-3p | CAAAAAUCUCAAUUACUUUUGC | |
| miR-7-5p | UGGAAGACUAGUGAUUUUGUUGUU | |
| miR-12120 | UAAGGAACGCGGGGCCUUGGUAGAGC | |
| miR-6875-3p | AUUCUUCCUGCCCUGGCUCCAU | |
| miR-6888-5p | AAGGAGAUGCUCAGGCAGAU | |
| miR-3118 | UGUGACUGCAUUAUGAAAAUUCU | |
| U6-F | CTCGCTTCGGCAGCACA | |
| U6-R | AACGCTTCACGAATTTGCGT | |
